# Coptidis Rhizoma for Obesity, Diabetes, and Dyslipidemia: An Integrated Meta-Analysis and In Silico Study

**DOI:** 10.3390/ijms27188109

**Published:** 2026-09-11

**Authors:** Chae-Yeon Kang, Yeon-Joo Yoo, Ji-Han Kim, Seung-Hoon Yoo, Hyun-Joo Kim, Min-Seong Lee, Byung-Cheol Lee

**Affiliations:** 1 Department of Clinical Korean Medicine, Graduate School, Kyung Hee University, 26, Kyungheedae-ro, Dongdaemun-gu, Seoul 02447, Republic of Korea; cykang@khu.ac.kr (C.-Y.K.); alice98113@khu.ac.kr (Y.-J.Y.); ilpc21@khu.ac.kr (J.-H.K.); ysh9306@khu.ac.kr (S.-H.Y.); gryee1@khu.ac.kr (H.-J.K.); min6691@khu.ac.kr (M.-S.L.); 2 Department of Nephrology & Endocrinology, Kyung Hee University Korean Medicine Hospital, Kyung Hee Medical Center, 23, Kyungheedae-ro, Dongdaemun-gu, Seoul 02447, Republic of Korea

**Keywords:** *Coptidis chinensis*, berberine, metabolic syndrome, type 2 diabetes mellitus, dyslipidemia, systematic review, network pharmacology, molecular docking

## Abstract

Obesity, diabetes, and dyslipidemia form an interconnected metabolic disease cluster driven by insulin resistance and chronic inflammation. This study evaluated the clinical efficacy and multi-target mechanisms of Coptidis Rhizoma for these metabolic disorders through an integrated systematic review, meta-analysis of randomized controlled trials (RCTs), network pharmacology, and molecular docking. Meta-analysis of 19 RCTs (n = 1718) demonstrated that Coptidis-containing formulations, primarily as adjunctive therapy, significantly improved primary clinical endpoints across all three metabolic conditions: body mass index (BMI: SMD = −0.72, 95% CI [−1.00, −0.44], *p* < 0.00001), glycated hemoglobin (HbA1c: SMD = −0.77, 95% CI [−1.07, −0.47], *p* < 0.00001), and low-density lipoprotein cholesterol (LDL-C: SMD = −1.10, 95% CI [−1.57, −0.62], *p* < 0.00001). Network analysis revealed shared targets enriched in lipid metabolism, inflammatory signaling, and PI3K-Akt/MAPK cascades. Molecular docking demonstrated favorable structural compatibility and predicted docking scores (ranging from −7.1 to −9.5 kcal/mol) of berberine toward key hub proteins, including PIK3CA, JAK2, MAOA, and PARP1. Consequently, Coptidis Rhizoma provides tangible clinical benefits across interconnected metabolic outcomes via the multi-target regulation of inflammatory and insulin signaling pathways. Clinically, Coptidis-containing formulations show promise as a complementary adjunct to conventional metabolic therapies, though future large-scale, standardized RCTs remain necessary to confirm long-term safety and optimize dosing regimens.

## 1. Introduction

Obesity, diabetes, and dyslipidemia form an interconnected cluster of metabolic disorders that together impose a substantial burden on global healthcare systems [1]. These conditions frequently co-occur, with obesity-related complications often clustering together rather than presenting independently [2,3] and individuals with obesity-associated diabetes commonly show a higher burden of dyslipidemia as well, as evidenced by the fact that combined control of glycemic, blood pressure, and lipid targets is achieved in only a minority of these patients [4]. Rather than progressing as isolated clinical entities, these metabolic abnormalities interact through overlapping pathophysiological processes. Visceral adiposity contributes to chronic, low-grade systemic inflammation, which in turn can impair peripheral insulin signaling and disrupt systemic lipid homeostasis [5]. The resulting insulin resistance is associated with hepatic overproduction of atherogenic lipoproteins and triglycerides, along with progressive pancreatic beta-cell dysfunction and the development of type 2 diabetes [6]. Given this metabolic interplay, therapeutic strategies capable of addressing multiple metabolic abnormalities at once may hold particular value in managing these interconnected conditions [7].

Although conventional pharmacotherapies, including GLP-1 receptor agonists (GLP-1RAs), statins, and metformin, remain central to the management of obesity, diabetes, and dyslipidemia, their use is not without drawbacks. Treatment-specific adverse effects, tolerability concerns, and, in some settings, substantial long-term costs can all limit their use [8,9]. Gastrointestinal adverse effects are common with GLP-1RAs. Statin-associated muscle symptoms and metformin-related gastrointestinal intolerance have also been reported to affect treatment persistence in some patients [10,11,12]. As combination and long-term pharmacotherapy become more common, therapeutic and adjunctive strategies capable of concurrently addressing multiple metabolic abnormalities without compounding this tolerability burden have attracted increasing clinical interest.

Coptidis Rhizoma (Huang Lian), the dried rhizome of *Coptis chinensis* Franch., has long been used in East Asian medicine, particularly for conditions described within the traditional concepts of “heat” and “dampness” [13]. Experimental studies report metabolic and anti-inflammatory activities of Coptidis Rhizoma and its constituents, including effects on glucose metabolism, insulin sensitivity, lipid metabolism, and inflammatory signaling [14,15,16]. It is used both as a single herb and as a component of multi-herb formulations in traditional medical practice [17], and Coptidis-containing formulations have been clinically investigated for metabolic disorders. Despite these promising experimental findings and widespread clinical use, a rigorous, high-quality systematic evaluation synthesizing the clinical efficacy of Coptidis-containing formulations across the triad of obesity, diabetes, and dyslipidemia is still lacking.

To address the intrinsic complexity of herbal medicines, integrating clinical evidence synthesis with network-based computational analysis has increasingly emerged as a robust methodological framework [18,19,20]. While systematic reviews and meta-analyses establish high-level clinical evidence regarding the overall therapeutic efficacy and safety of whole herbs or complex multi-herb formulations, they are inherently limited in elucidating the fine-grained, multi-target molecular pathways involved. Conversely, network pharmacology and molecular docking provide powerful systems-biology tools to map multi-component and multi-target interactions at the molecular level, generating mechanistic hypotheses that contextualize and substantiate observed clinical outcomes. Because modern pharmacological investigations of Coptidis Rhizoma have predominantly characterized berberine as its principal bioavailable alkaloid accounting for its primary metabolic actions [17], berberine serves as an optimal, well-defined representative constituent and molecular entry point for cross-disease network modeling. Furthermore, because herbal medicines in real-world clinical practice are frequently administered as adjunctive regimens alongside standard pharmacotherapies, evaluating both pragmatic add-on clinical trials and underlying molecular pathways provides a translational bridge from bedside efficacy to bench-level mechanism. By linking clinical trial synthesis with network pharmacology, this integrated strategy bridges the gap between macro-level clinical phenotypes and micro-level molecular mechanisms across the triad of obesity, diabetes, and dyslipidemia.

Accordingly, this study aimed to provide a comprehensive, multi-dimensional evaluation of Coptidis-containing formulations across the interconnected cluster of obesity, type 2 diabetes mellitus, and dyslipidemia. Specifically, the first objective was to systematically evaluate the clinical efficacy and safety of Coptidis-containing formulations on anthropometric, glycemic, and lipid parameters through a systematic review and meta-analysis of randomized controlled trials (RCTs). The second objective was to elucidate the underlying multi-target molecular mechanisms of its representative alkaloid, berberine, by identifying common, interconnected disease targets and key biological pathways through network pharmacology and molecular docking analyses. By synthesizing macroscopic clinical evidence with microscopic systems-level pharmacology, this study establishes both a rigorous evidence base and a plausible mechanistic framework for the therapeutic application of Coptidis Rhizoma in metabolic syndrome.

## 2. Results

### 2.1. General Description of the Included Studies

**Figure 1:**
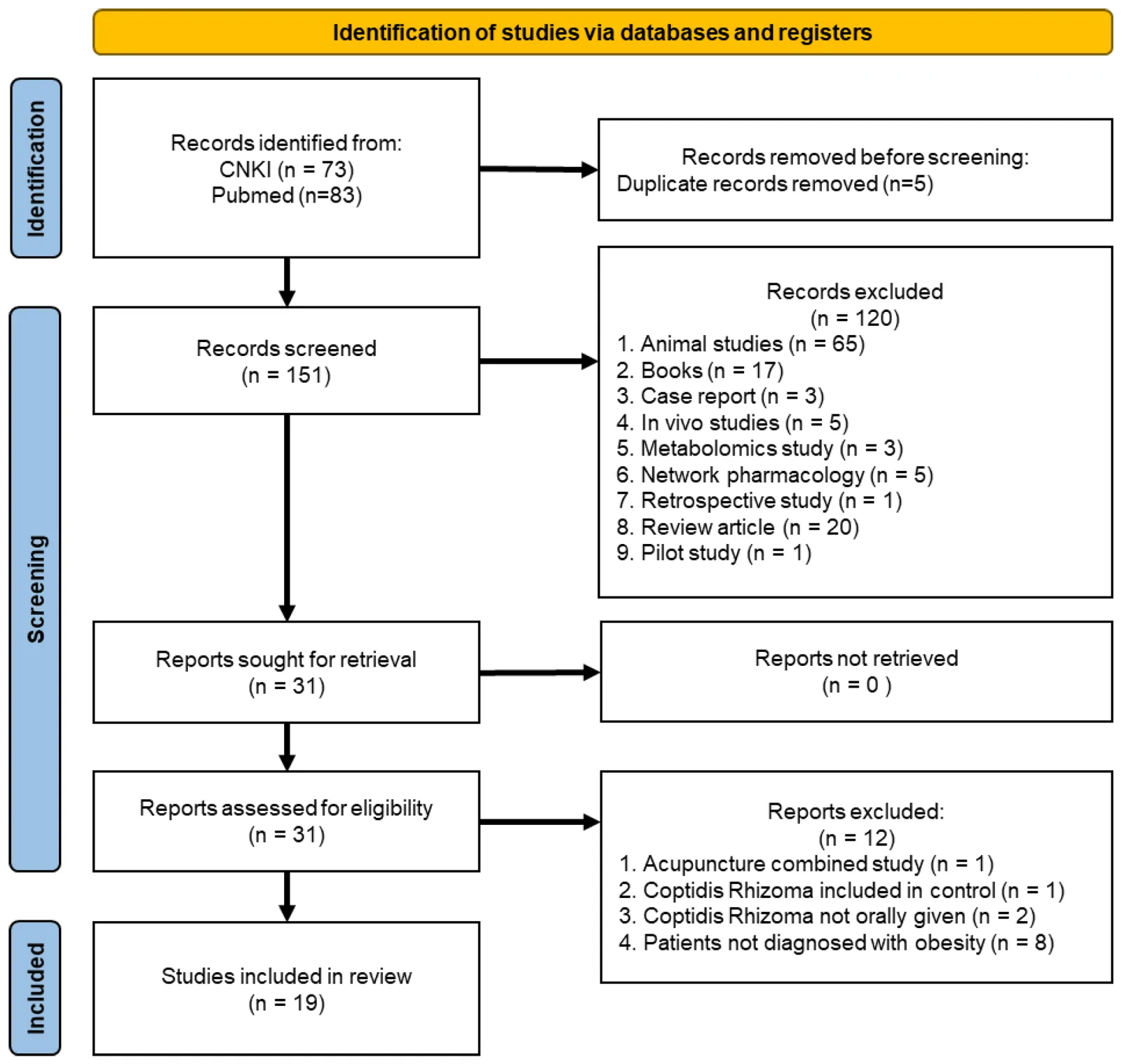
Meta-analysis flow diagram.

A total of 156 records were identified (China National Knowledge Infrastructure [CNKI], n = 73; PubMed, n = 83), of which 151 remained after removal of duplicates (n = 5). Following title and abstract screening, 120 studies were excluded, primarily due to non-clinical design (animal studies, n = 65), non-original formats (books, n = 17; reviews, n = 20), or ineligible methodologies (in vivo studies, n = 5; metabolomics, n = 3; network pharmacology, n = 5; case reports, n = 3; retrospective study, n = 1; pilot study, n = 1). Full-text reports were sought for the remaining 31 records, and all 31 reports were successfully retrieved without any loss due to inaccessible or missing full texts (n = 0). These 31 studies underwent full-text assessment, of which 12 were excluded because of combined acupuncture interventions (n = 1), inclusion of Coptidis Rhizoma in the control group (n = 1), non-oral administration (n = 2), or absence of a defined obesity diagnosis (n = 8). Nineteen RCTs met the inclusion criteria and were included in the final analysis; all were conducted in China. Study selection is summarized in Figure 1, and detailed characteristics are presented in Table 1. The included trials evaluated outcomes across three domains: obesity-related indices, glycemic parameters, and lipid profiles.

### 2.2. Results of the Risk of Bias Assessment

**Figure 2:**
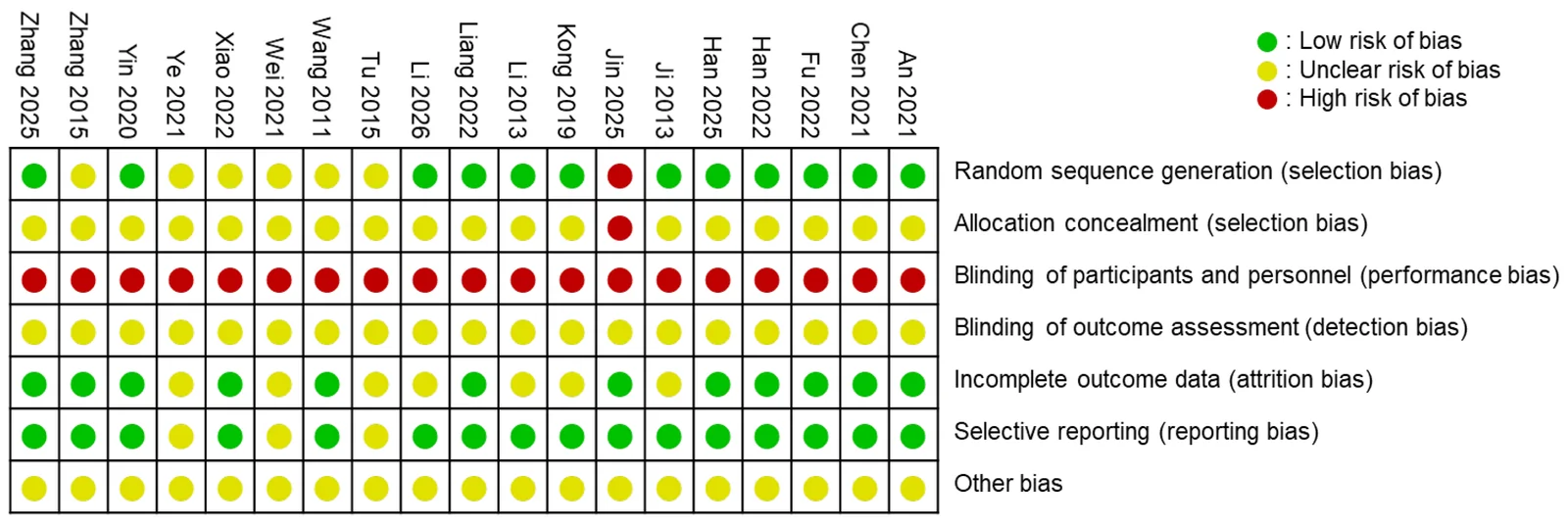
Risk of bias on selected studies [21,22,23,24,25,26,27,28,29,30,31,32,33,34,35,36,37,38,39].

Methodological quality assessment of the included randomized controlled trials revealed that the principal methodological concerns across studies were the lack of blinding of participants and personnel (performance bias) and incomplete reporting of allocation concealment. The detailed study-level risk of bias judgements are illustrated in Figure 2.

The certainty of evidence evaluated using the Grading of Recommendations Assessment, Development and Evaluation (GRADE) approach ranged from moderate to very low across all synthesized endpoints (Table A2), with risk of bias and substantial statistical heterogeneity serving as the primary downgrading factors. Specifically, the certainty of evidence was graded as low for body mass index (BMI), body weight (BW), FPG (fasting plasma glucose), 2hPG (2 h postprandial glucose), glycated hemoglobin (HbA1c), total cholesterol (TC), triglycerides (TG), low-density lipoprotein cholesterol (LDL-C), and high-density lipoprotein cholesterol (HDL-C), and very low for waist circumference (WC). In contrast, homeostatic model assessment of insulin resistance (HOMA-IR) was graded as moderate certainty owing to consistent effect estimates across trials.

### 2.3. Meta-Analysis of Intervention Effects

#### 2.3.1. Efficacy on Obesity-Related Parameters

**Figure 3:**
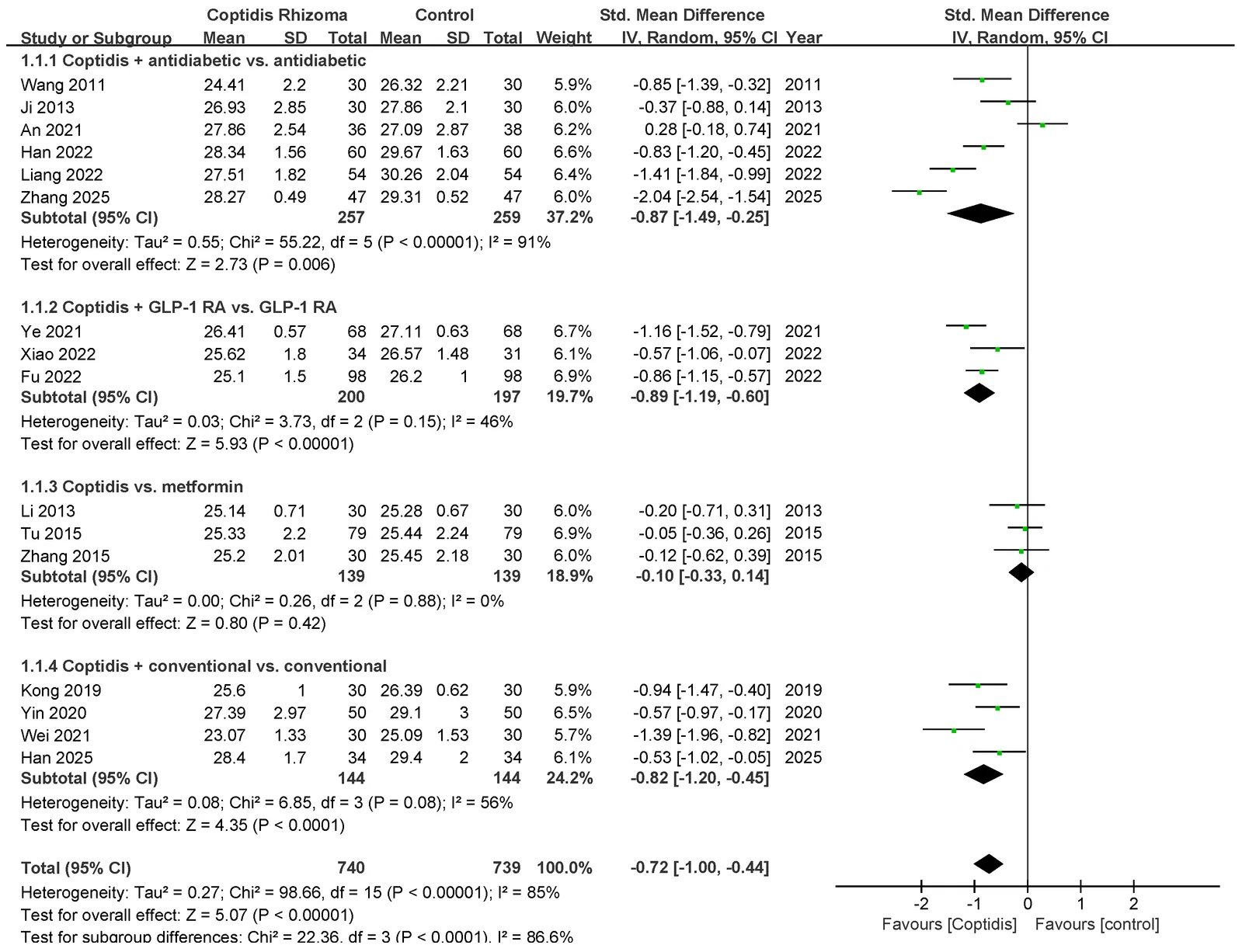
Complete data of body mass index (BMI) [22,23,25,26,27,28,29,31,32,33,34,35,36,37,38,39]. *Green squares indicate point estimates (SMDs) with size proportional to statistical weight; horizontal lines represent 95% CIs; black diamonds denote pooled effect sizes; and the vertical solid line represents the line of no effect*.

BMI was evaluated as the primary clinical outcome to assess the anti-obesity efficacy of Coptidis-containing formulations. A comprehensive pooled analysis of 16 RCTs demonstrated that Coptidis-based interventions led to a statistically significant reduction in BMI compared to the control groups, yielding a pooled standardized mean difference (SMD) of −0.72 (95% CI: [−1.00, −0.44], *p* < 0.00001; Table 2, Figure 3). Although substantial overall heterogeneity was detected (I^2^ = 85%, *p* < 0.00001), subgroup analyses revealed high consistency and marked therapeutic benefits, particularly when Coptidis-containing formulations were combined with GLP-1 receptor agonists (3 RCTs, SMD: −0.89, 95% CI: [−1.19, −0.60], I^2^ = 46%) or conventional treatments (4 RCTs, SMD: −0.82, 95% CI: [−1.20, −0.45], I^2^ = 56%).

**Figure A1:**
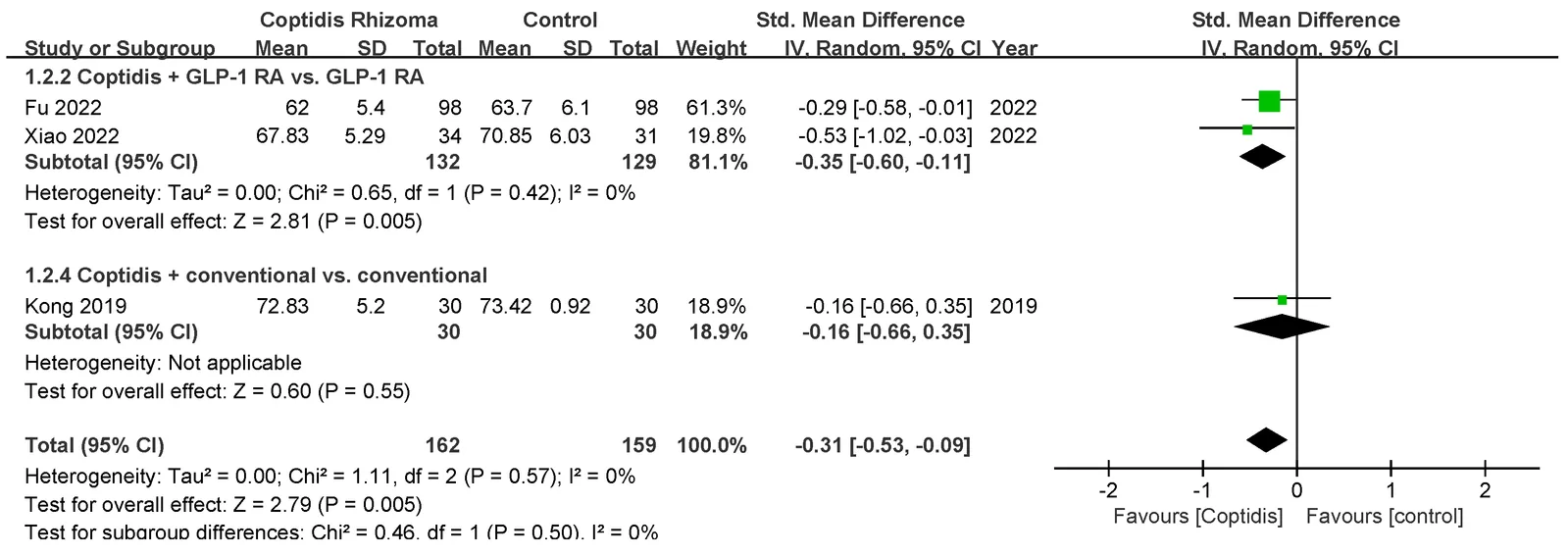
Complete data of body weight [26,28,34]. *Symbols and graphical elements are defined as in Figure 3*.

**Figure A2:**
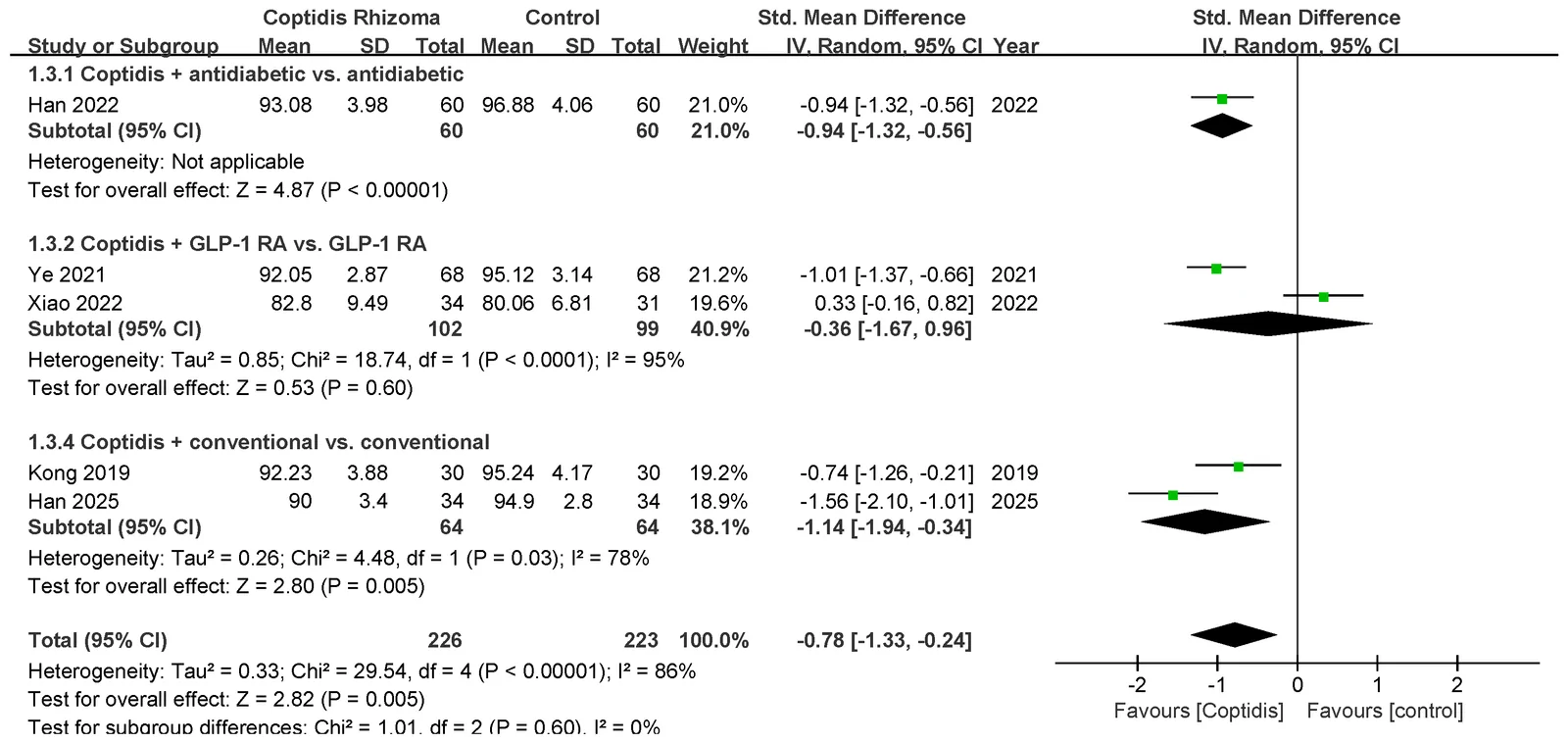
Complete data of waist circumference [23,27,28,29,34]. *Symbols and graphical elements are defined as in Figure 3*.

In addition to BMI, other obesity-related parameters, including BW and WC, were evaluated as secondary outcomes. A total of 3 RCTs reported data on BW. The pooled analysis demonstrated that Coptidis-containing formulations significantly reduced body weight compared to the control group, with an overall SMD of −0.31 (95% CI: [−0.53, −0.09], *p* = 0.005) and no evidence of between-study heterogeneity (I^2^ = 0%, *p* = 0.57; Figure A1, Table A4). Furthermore, 5 RCTs evaluated changes in WC. The adjunctive use of Coptidis-based interventions led to a significant decrease in WC, yielding an overall SMD of −0.78 (95% CI: [−1.33, −0.24], *p* = 0.005; Figure A2, Table A5).

#### 2.3.2. Efficacy on Glycemic Control and Insulin Sensitivity

**Figure 4:**
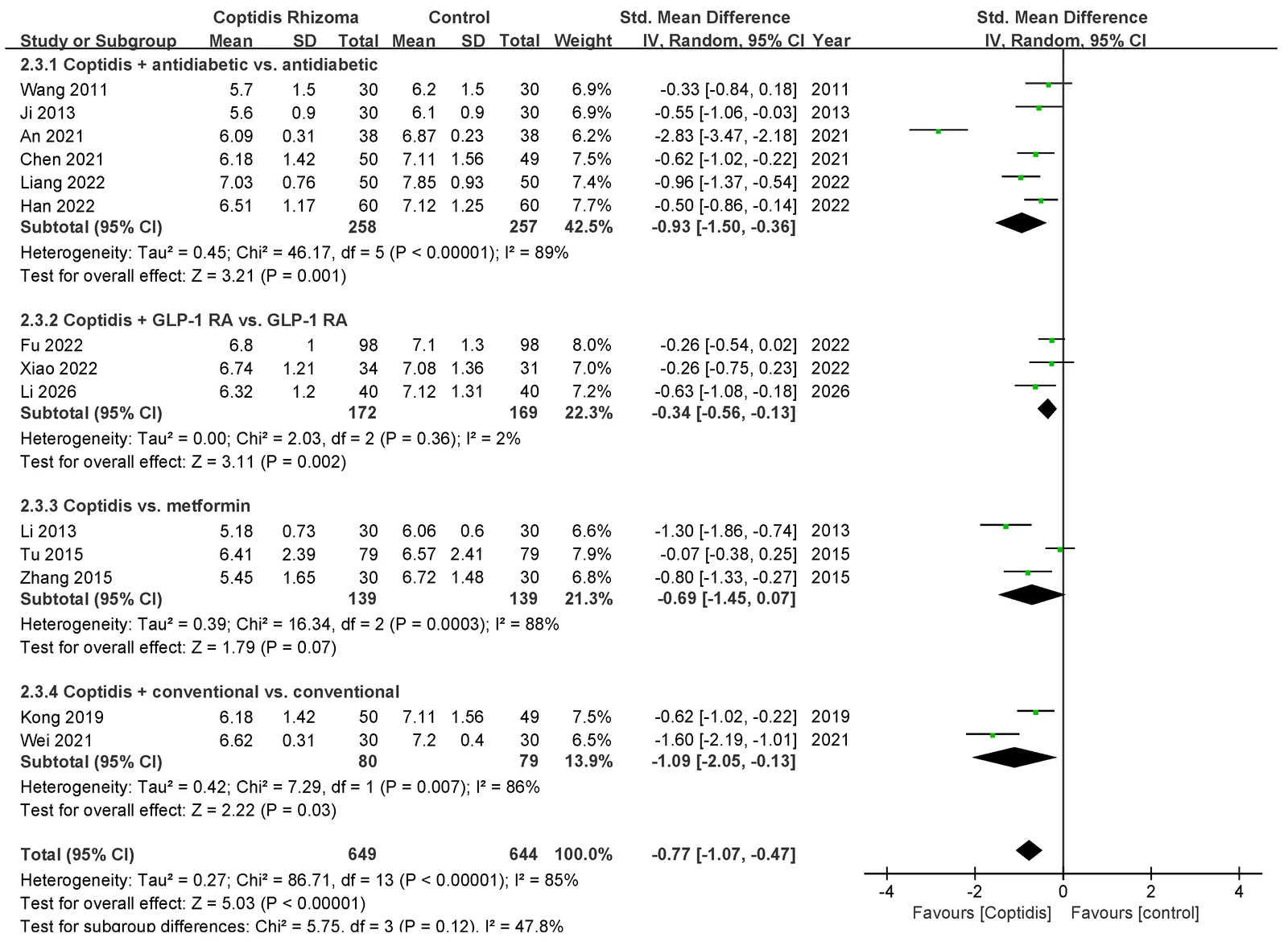
Complete data of glycated hemoglobin [21,23,25,26,28,30,31,32,34,35,36,37,38,39]. *Symbols and graphical elements are defined as in Figure 3*.

HbA1c was designated as the primary endpoint to rigorously evaluate long-term glycemic control, as it reflects stable, cumulative glucose homeostasis. Quantitative synthesis of 14 RCTs revealed that Coptidis-containing formulations achieved a highly significant post-treatment decline in HbA1c levels relative to control therapies, with an overall pooled SMD of −0.77 (95% CI: [−1.07, −0.47], *p* < 0.00001; Table 3, Figure 4). Notably, the adjunctive use of Coptidis with GLP1RAs demonstrated an exceptionally robust and homogenous therapeutic profile (3 RCTs, SMD: −0.34, 95% CI: [−0.56, −0.13], I^2^ = 2%, *p* = 0.36), highlighting its clinical value as a highly dependable combination therapy.

**Figure A3:**
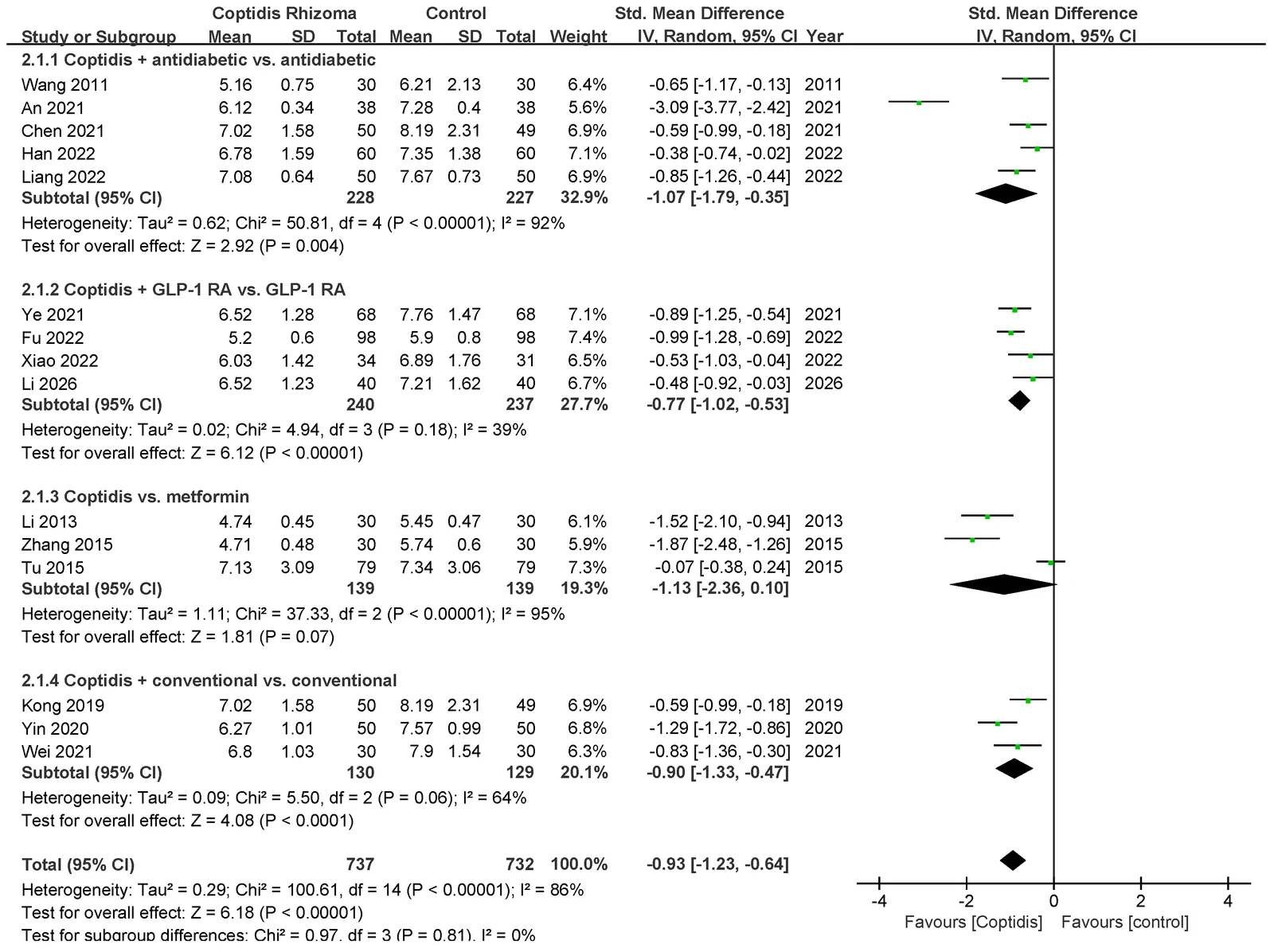
Complete data of fasting plasma glucose [21,25,26,27,28,29,30,31,32,33,34,35,36,38,39]. *Symbols and graphical elements are defined as in Figure 3*.

**Figure A4:**
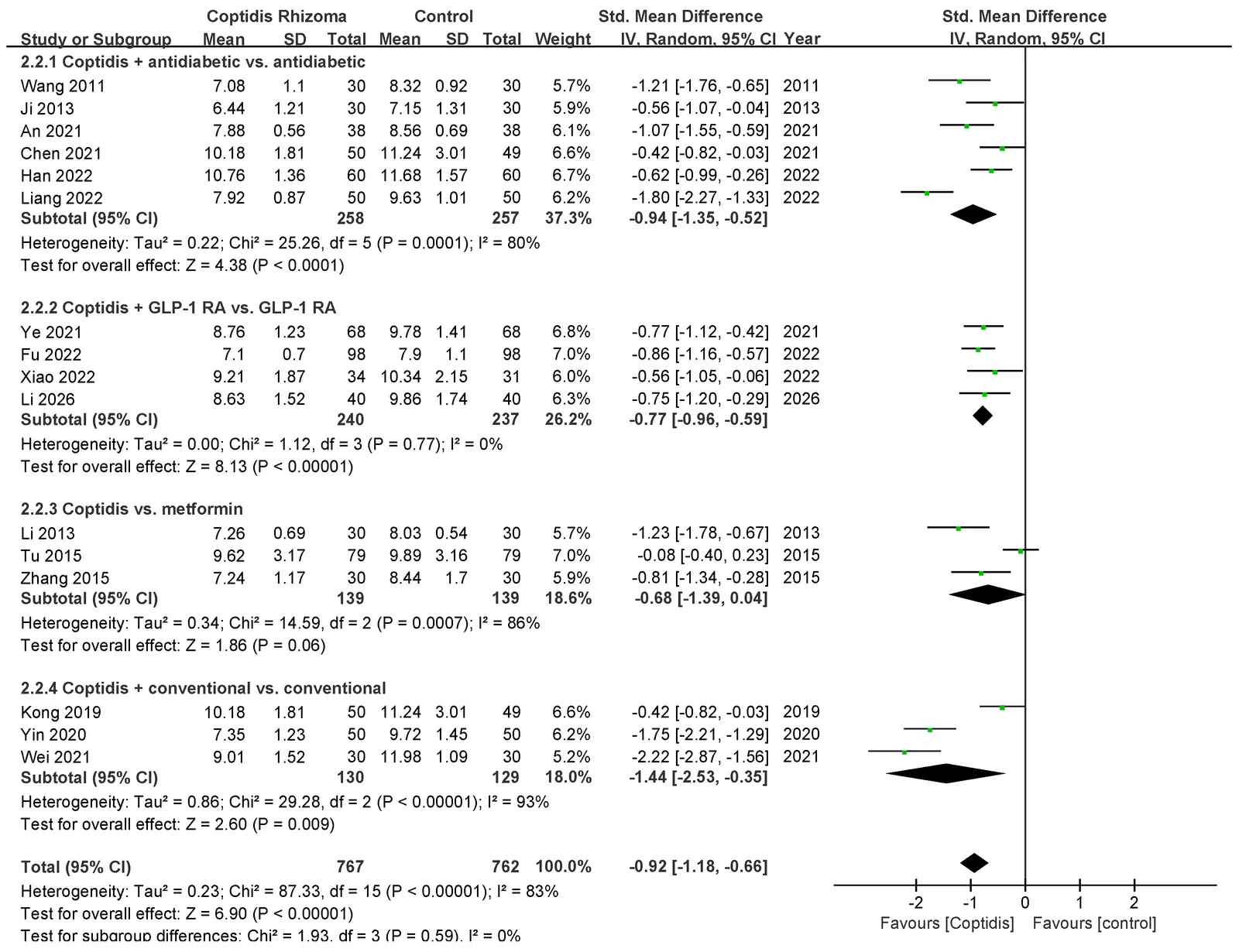
Complete data of 2 h postprandial glucose [21,25,26,27,28,29,30,31,32,33,34,35,36,37,38,39]. *Symbols and graphical elements are defined as in Figure 3*.

**Figure A5:**
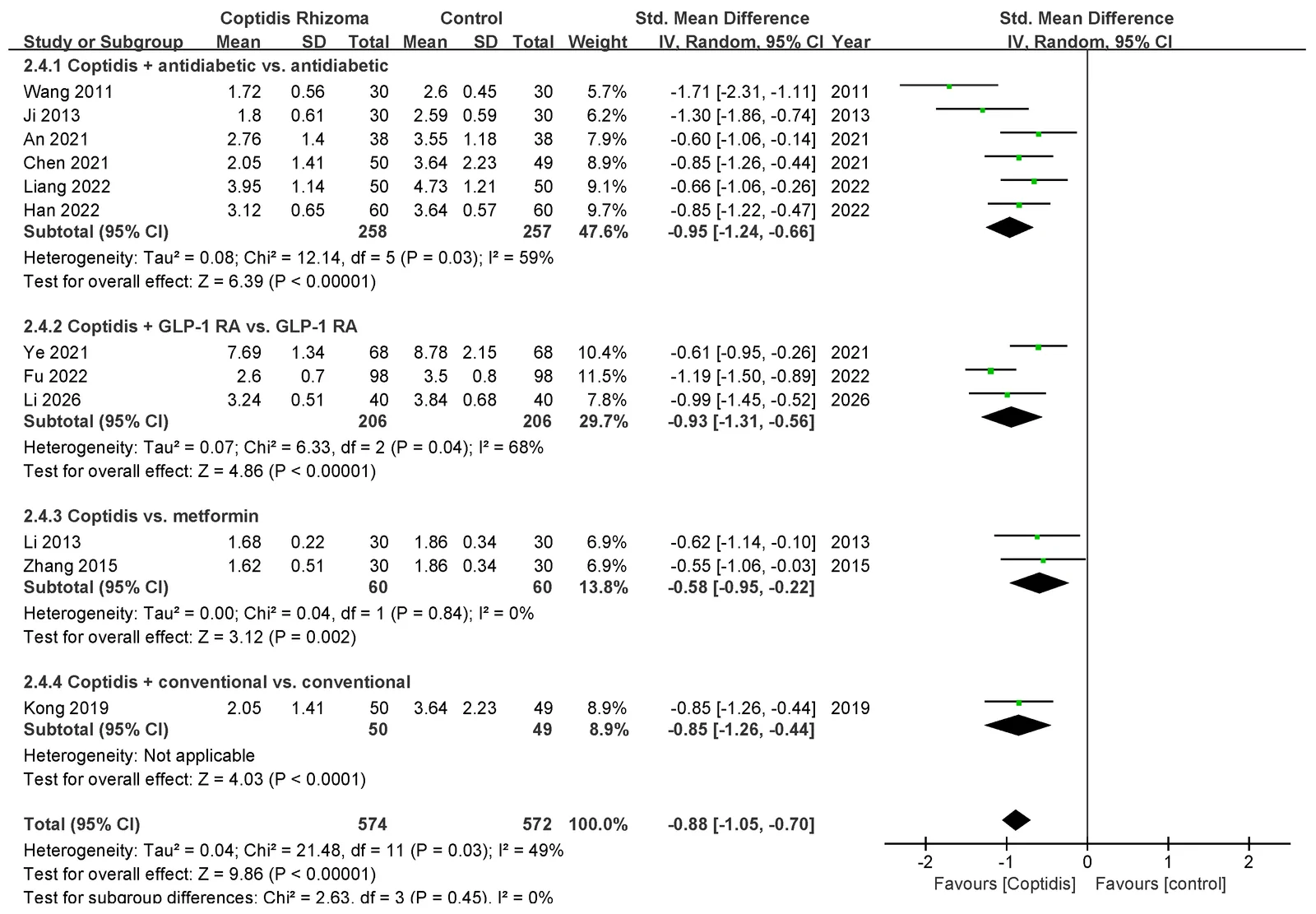
Complete data of homeostatic model assessment of insulin resistance [21,23,25,26,29,30,31,33,34,35,37,38,39]. *Symbols and graphical elements are defined as in Figure 3*.

To provide a comprehensive assessment of glycemic control, FPG, 2hPG, and HOMA-IR were analyzed alongside HbA1c. FPG data were synthesized from 15 RCTs, showing a significant reduction in favor of the Coptidis group (overall SMD: −0.93, 95% CI: [−1.23, −0.64], *p* < 0.00001; Figure A3, Table A6). Similarly, the pooled analysis of 16 RCTs for 2hPG revealed a marked therapeutic benefit (overall SMD: −0.92, 95% CI: [−1.18, −0.66], *p* < 0.00001; Figure A4, Table A7). For insulin resistance, 12 RCTs reporting HOMA-IR indices were evaluated. Coptidis-containing formulations significantly ameliorated insulin resistance compared to conventional therapies alone, demonstrating a pooled SMD of −0.88 (95% CI: [−1.05, −0.70], *p* < 0.00001; Figure A5, Table A8). Substantial between-study heterogeneity was noted for both FPG (I^2^ = 86%) and 2hPG (I^2^ = 83%), whereas HOMA-IR exhibited a relatively moderate and acceptable heterogeneity profile (I^2^ = 49%, *p* = 0.03).

#### 2.3.3. Efficacy on Lipid Profiles

**Figure 5:**
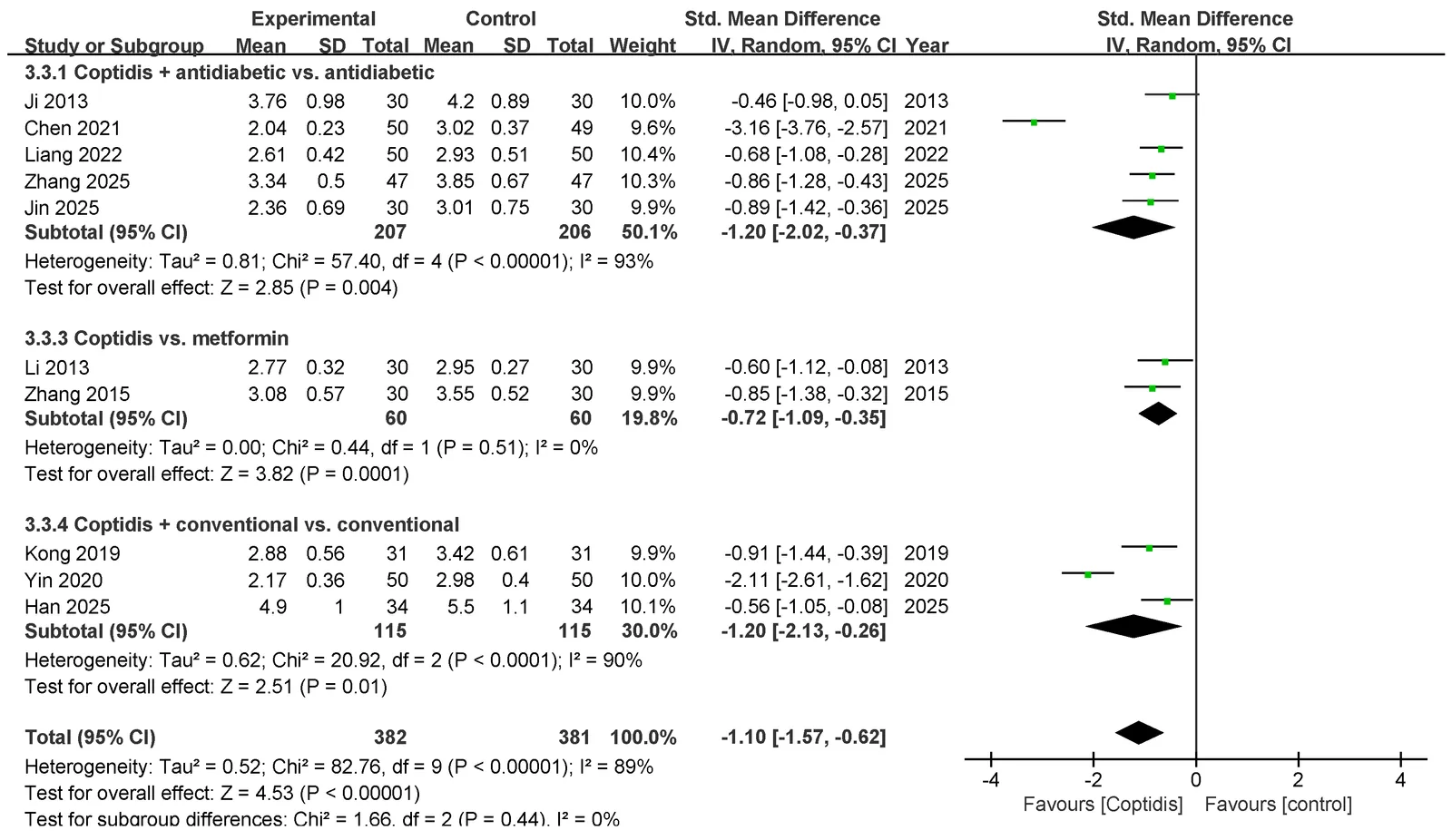
Complete data of low-density lipoprotein cholesterol [22,24,25,27,30,33,34,35,37,38]. *Symbols and graphical elements are defined as in Figure 3*.

LDL-C was selected as the primary outcome for the lipid domain, given its direct clinical correlation with atherosclerosis and overall cardiometabolic risk. Based on the pooled analysis of 10 RCTs, Coptidis-based interventions exerted a profound lipid-lowering efficacy, resulting in a marked and statistically significant reduction in LDL-C compared to control groups (SMD: −1.10, 95% CI: [−1.57, −0.62], *p* < 0.00001; Table 4, Figure 5). When Coptidis was used specifically as an adjunct to metformin, the therapeutic effect was exceptionally stable and uniform across trials (2 RCTs, SMD: −0.72, 95% CI: [−1.09, −0.35], I^2^ = 0%, *p* = 0.51).

**Figure A6:**
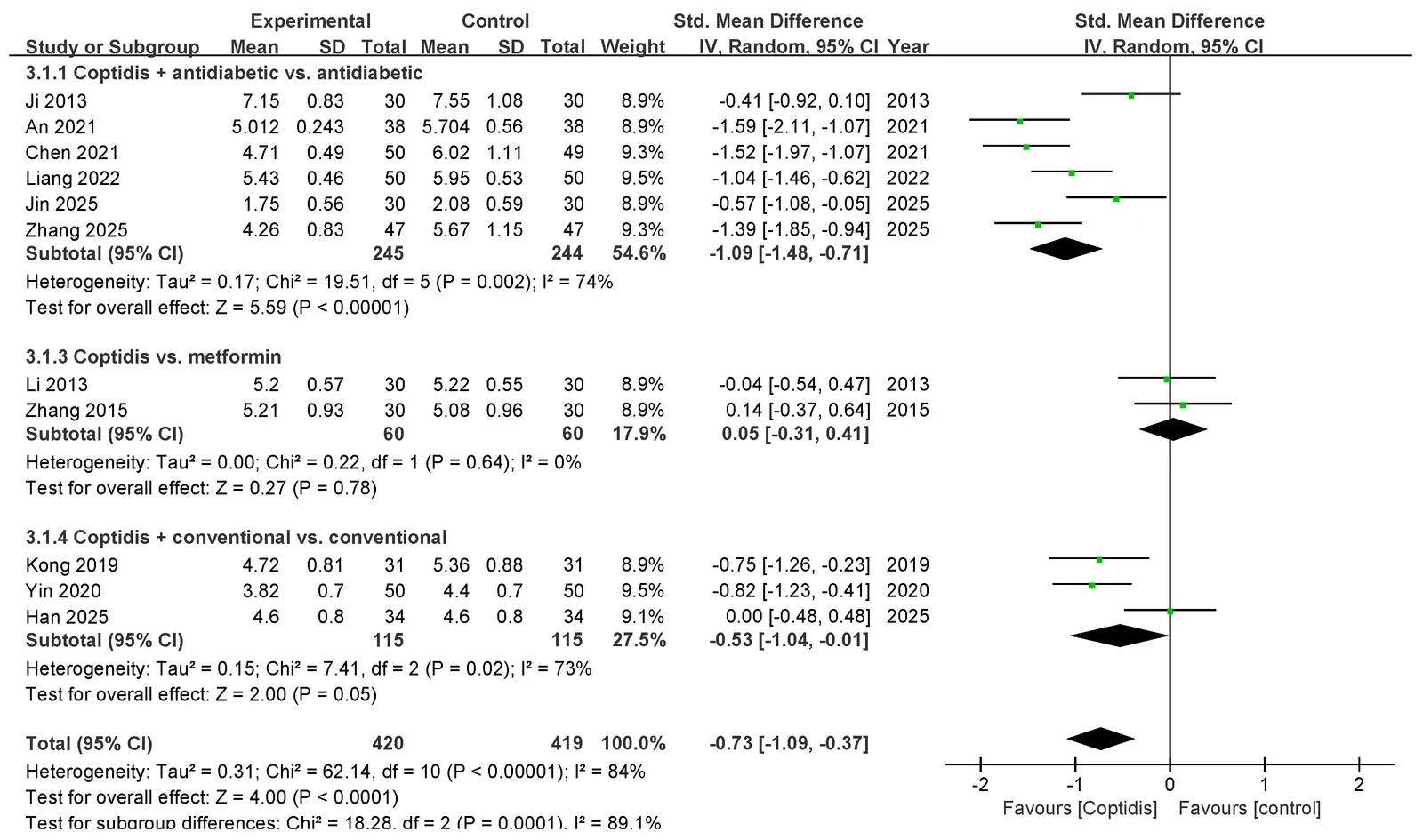
Complete data of total cholesterol [22,24,25,27,30,31,33,34,35,37,38]. *Symbols and graphical elements are defined as in Figure 3*.

**Figure A7:**
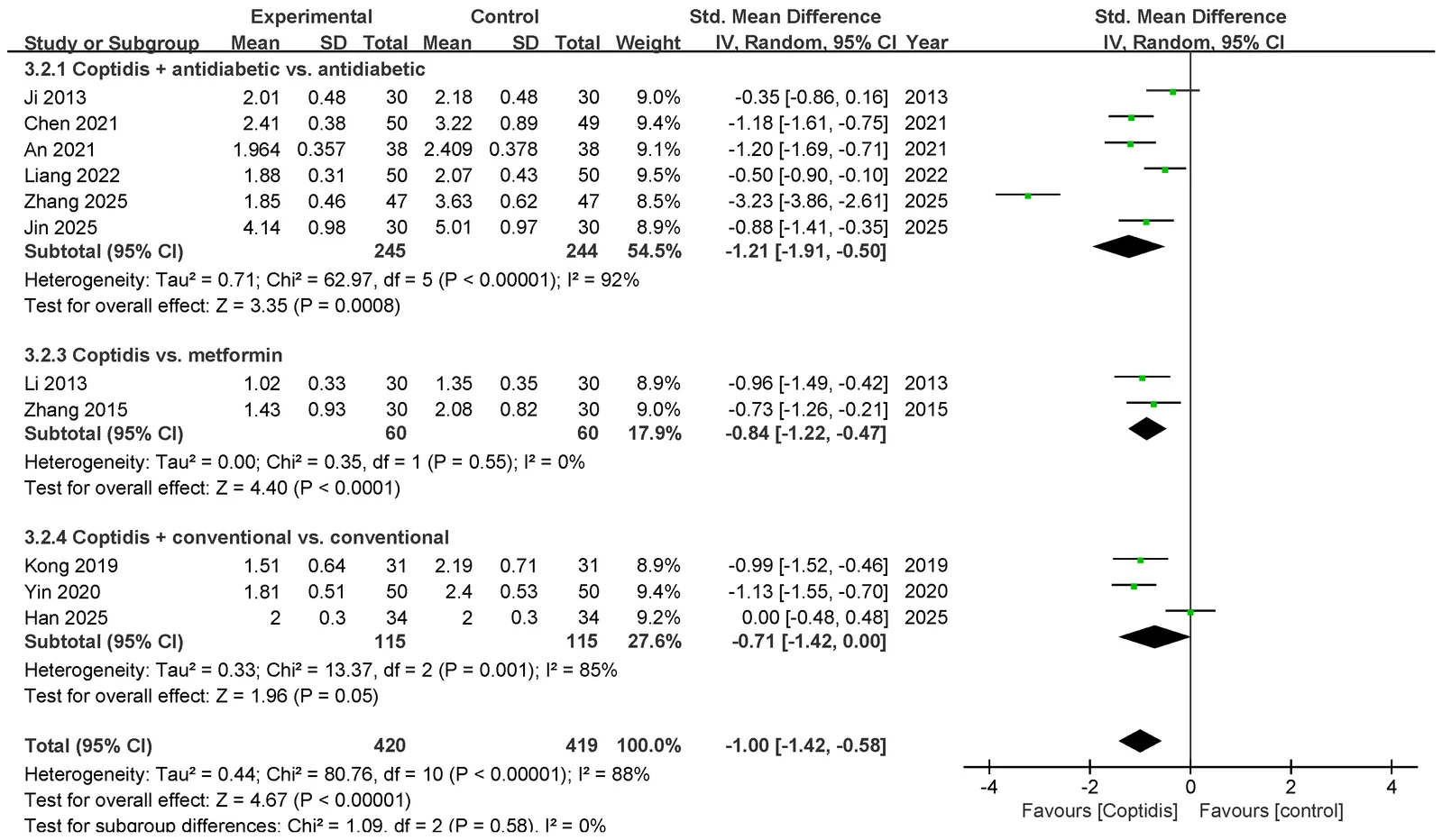
Complete data of triglycerides [22,23,24,25,27,30,31,33,34,35,37,38]. *Symbols and graphical elements are defined as in Figure 3*.

**Figure A8:**
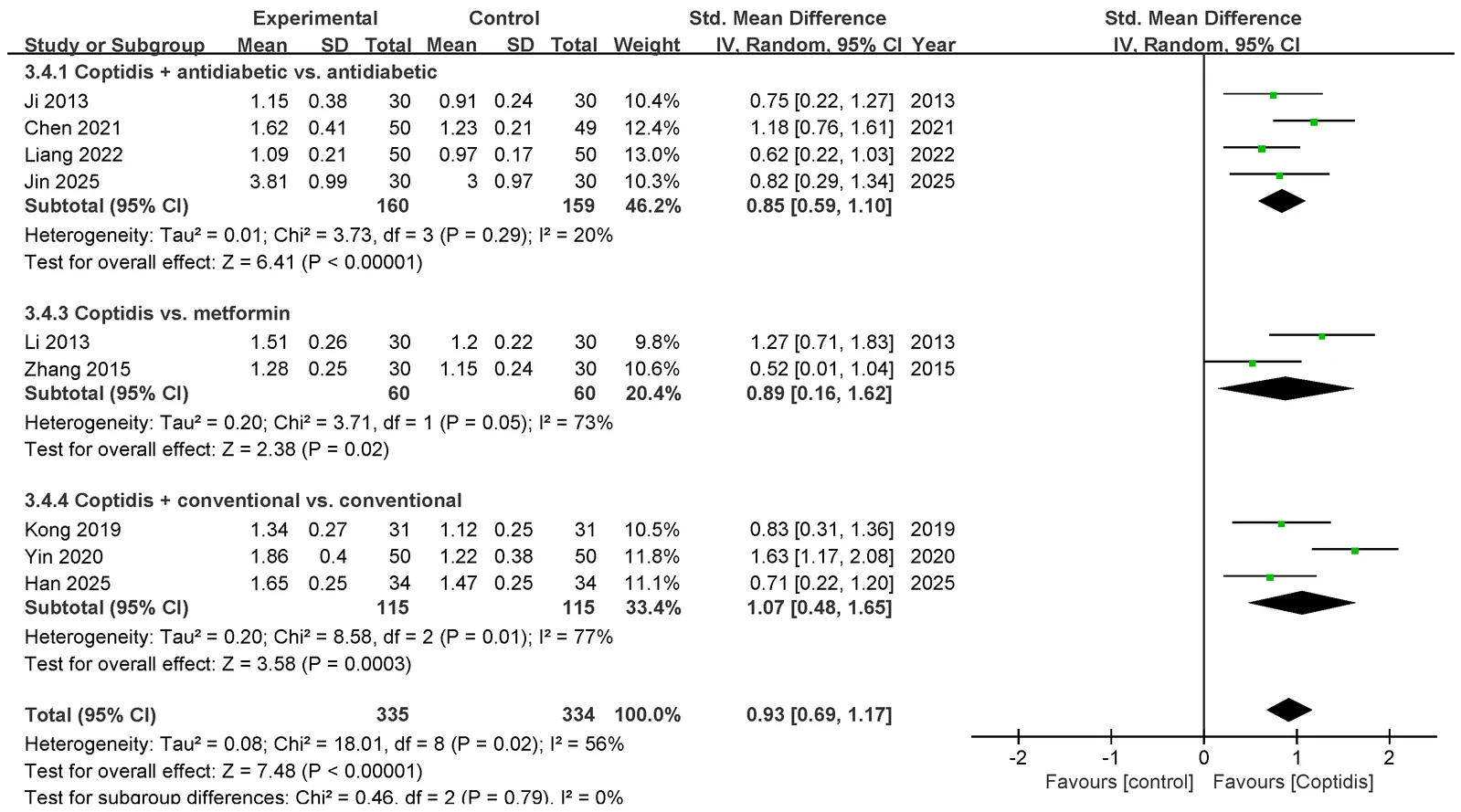
Complete data of high-density lipoprotein cholesterol [23,24,25,30,33,34,35,37,38]. *Symbols and graphical elements are defined as in Figure 3*.

Beyond LDL-C, the clinical efficacy of Coptidis-based interventions on the broader lipid profile was verified through pooled analyses of TC, TG, and HDL-C. For TC, data from 11 RCTs were synthesized, indicating a significant post-treatment decline compared to the control groups (overall SMD: −0.73, 95% CI: [−1.09, −0.37], *p* < 0.0001; Figure A6, Table A9). Analysis of 11 RCTs evaluating TG levels also demonstrated a significant therapeutic effect, with a pooled SMD of −1.00 (95% CI: [−1.42, −0.58], *p* < 0.00001; Figure A7, Table A10). Lastly, 9 RCTs reported favorable changes in HDL-C levels. Coptidis-containing formulations significantly elevated HDL-C profiles, yielding a combined SMD of 0.93 (95% CI: [0.69, 1.17], *p* < 0.00001; Figure A8, Table A11). High heterogeneity was observed in both TC (I^2^ = 84%) and TG (I^2^ = 88%) analyses, whilst the HDL-C subgroup data presented moderate heterogeneity (I^2^ = 56%, *p* = 0.02).

### 2.4. In Silico Network Pharmacology Analysis

#### 2.4.1. ADME Properties of Berberine from Coptidis Rhizoma

The absorption, distribution, metabolism, and excretion (ADME) properties of berberine obtained from the TCMSP database are summarized in Table 5. Berberine exhibited a molecular weight of 336.39, an oral bioavailability (OB) of 36.86%, a drug-likeness (DL) score of 0.78, a Caco-2 permeability of 1.24, a blood–brain barrier (BBB) permeability of 0.57, and a half-life of 6.57 h. These parameters met the standard screening criteria (OB ≥ 30%, Caco-2 permeability > −0.4, and DL ≥ 0.18).

#### 2.4.2. Identifying Overlapping Genes of Berberine and Metabolic Disease-Related Genes

The results showed that berberine shared overlapping targets with all three metabolic disease categories. Specifically, 94 overlapping genes were identified between berberine and obesity-related targets, 95 overlapping genes between berberine and diabetes-related targets, and 54 overlapping genes between berberine and dyslipidemia-related targets. These overlapping genes were considered candidate targets through which berberine may exert regulatory effects on obesity-, diabetes-, and dyslipidemia-related metabolic dysfunction.

**Figure 6:**
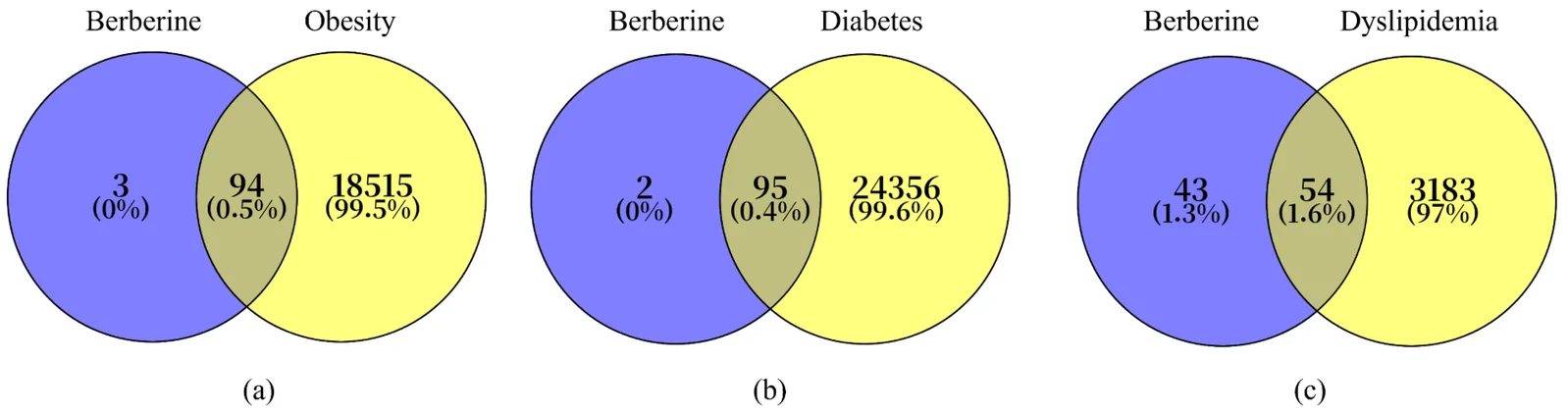
Overlapping target genes between berberine and metabolic disease-related genes. (**a**) Overlapping genes between berberine and obesity-related targets; (**b**) overlapping genes between berberine and diabetes-related targets; (**c**) overlapping genes between berberine and dyslipidemia-related targets.

The identified overlapping genes were subsequently used for protein–protein interaction (PPI) network construction, hub-gene analysis, and functional enrichment analyses, including Gene Ontology (GO) and Kyoto Encyclopedia of Genes and Genomes (KEGG) (Figure 6).

#### 2.4.3. Network Characteristics of Berberine-Associated Targets in Obesity, Diabetes, and Dyslipidemia

**Figure 7:**
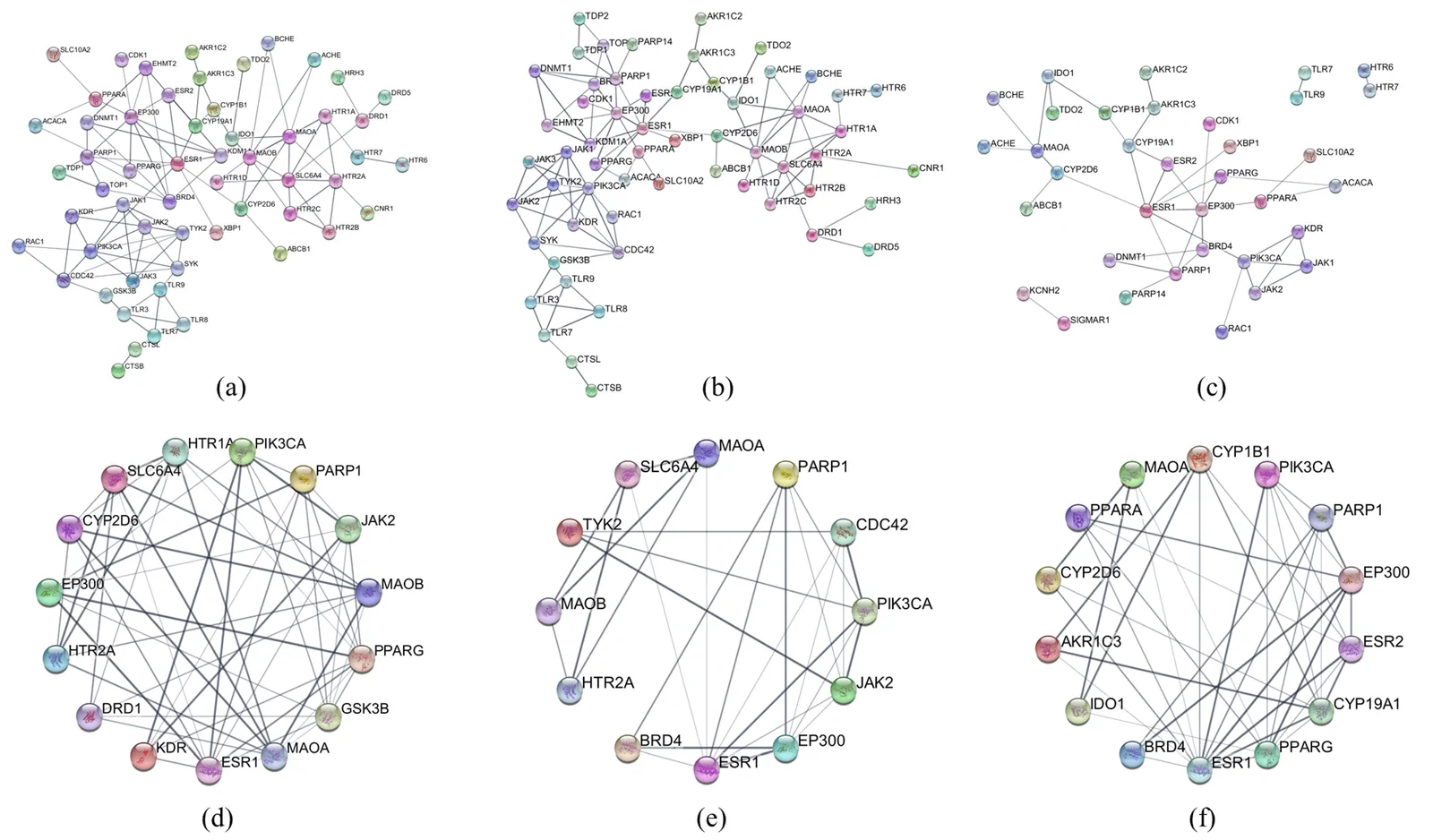
Network analysis of berberine-related targets associated with obesity, diabetes, and dyslipidemia. (**a**) STRING-based protein–protein interaction network of overlapping targets between berberine and obesity-related genes. (**b**) STRING-based protein–protein interaction network of overlapping targets between berberine and diabetes-related genes. (**c**) STRING-based protein–protein interaction network of overlapping targets between berberine and dyslipidemia-related genes. (**d**) Hub-gene subnetwork of berberine–obesity targets visualized using a circular layout. (**e**) Hub-gene subnetwork of berberine–diabetes targets visualized using a circular layout. (**f**) Hub-gene subnetwork of berberine–dyslipidemia targets visualized using a circular layout.

Topological analysis of the STRING-based PPI networks identified distinct hub-gene subnetworks for each disease condition (Figure 7a–c). Specifically, 15 hub genes were identified in the obesity network (Figure 7d), 12 in the diabetes network (Figure 7e), and 14 in the dyslipidemia network (Figure 7f). Among these, five targets—MAOA, PIK3CA, PARP1, EP300, and ESR1—were identified as common hub genes shared across all three disease-related subnetworks.

#### 2.4.4. GO and KEGG Pathway Enrichment Analysis

**Figure 8:**
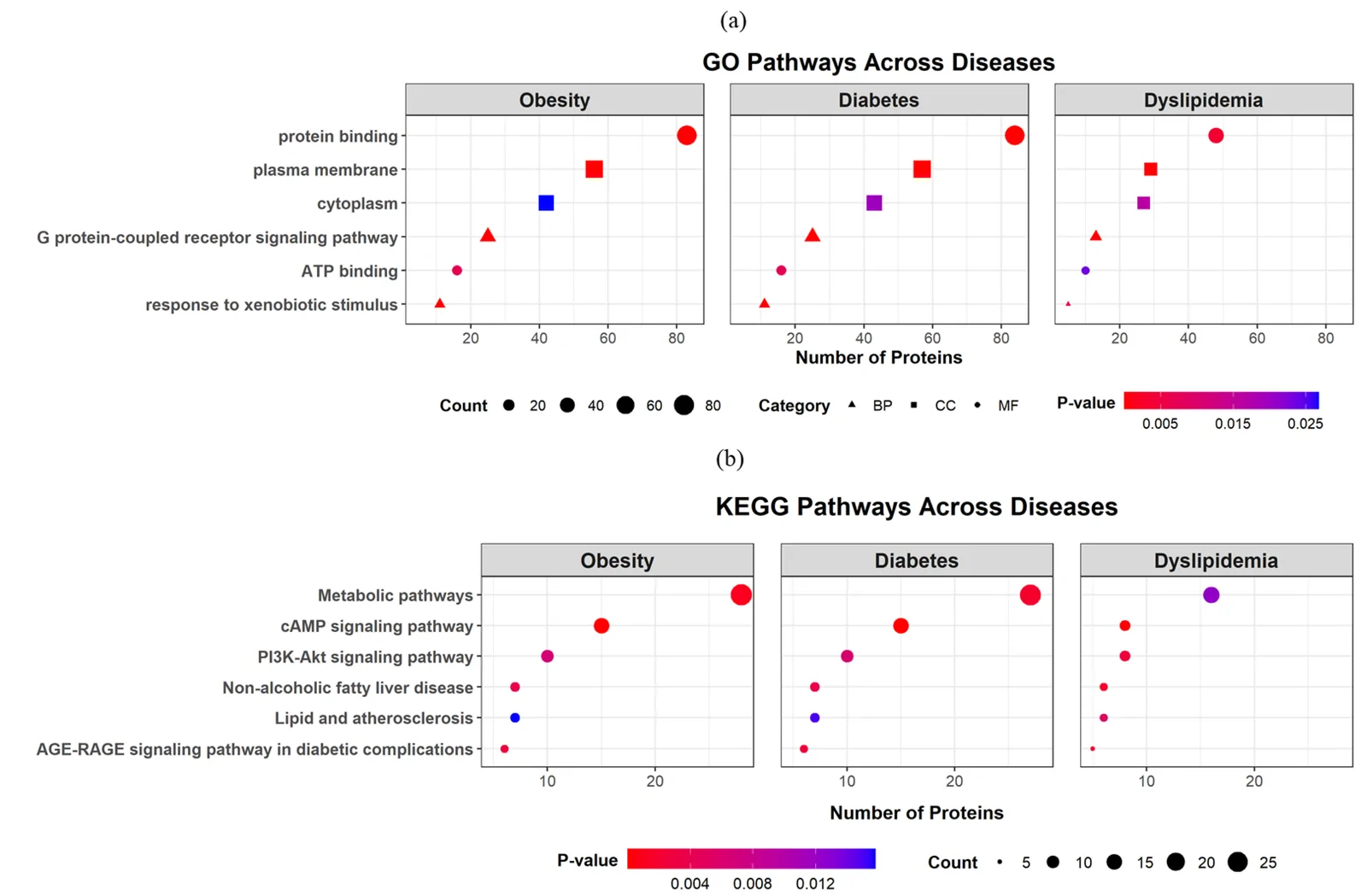
Comparative GO functional and KEGG pathway enrichment of berberine-related targets across obesity, diabetes, and dyslipidemia. (**a**) Representative GO terms commonly enriched across the three disease-related target sets, displaying the top shared terms across Biological Process (BP, triangles), Cellular Component (CC, squares), and Molecular Function (MF, circles). (**b**) Representative KEGG pathways commonly enriched across the three disease-related target sets. For both analyses, bubble size (Count) represents the number of target proteins, and bubble color indicates statistical significance (*p*-value).

GO and KEGG pathway enrichment analyses were performed to investigate the biological functions and signaling pathways associated with the overlapping targets between berberine and metabolic disease-related genes. To facilitate the molecular interconnections among the disorders and improve visual clarity, the analysis was focused on common, representative terms shared across obesity, diabetes, and dyslipidemia (Figure 8).

GO enrichment analysis revealed broadly consistent functional patterns across the three disease target sets (Figure 8a). Within the Biological Process (BP) category, G protein-coupled receptor (GPCR) signaling and response to xenobiotic stimulus were commonly enriched. In the Cellular Component (CC) category, plasma membrane and cytoplasm were prominently represented, whereas protein binding and ATP binding were the major Molecular Function (MF) terms shared across all three conditions.

KEGG enrichment analysis revealed six representative pathways commonly enriched across obesity, diabetes, and dyslipidemia: metabolic pathways, cAMP signaling pathway, PI3K-Akt signaling pathway, non-alcoholic fatty liver disease (NAFLD), lipid and atherosclerosis, and AGE-RAGE signaling pathway in diabetic complications (Figure 8b).

### 2.5. Molecular Docking Analysis

Molecular docking analysis was performed to investigate the binding interactions between berberine and key target proteins identified through the network pharmacology analysis. The selected target proteins included PIK3CA, RAC1, CHRM1, HTR6, HTR7, HTR2C, GSK3B, JAK2, MAOA, PARP1, EP300, and ESR1.

**Figure 9:**
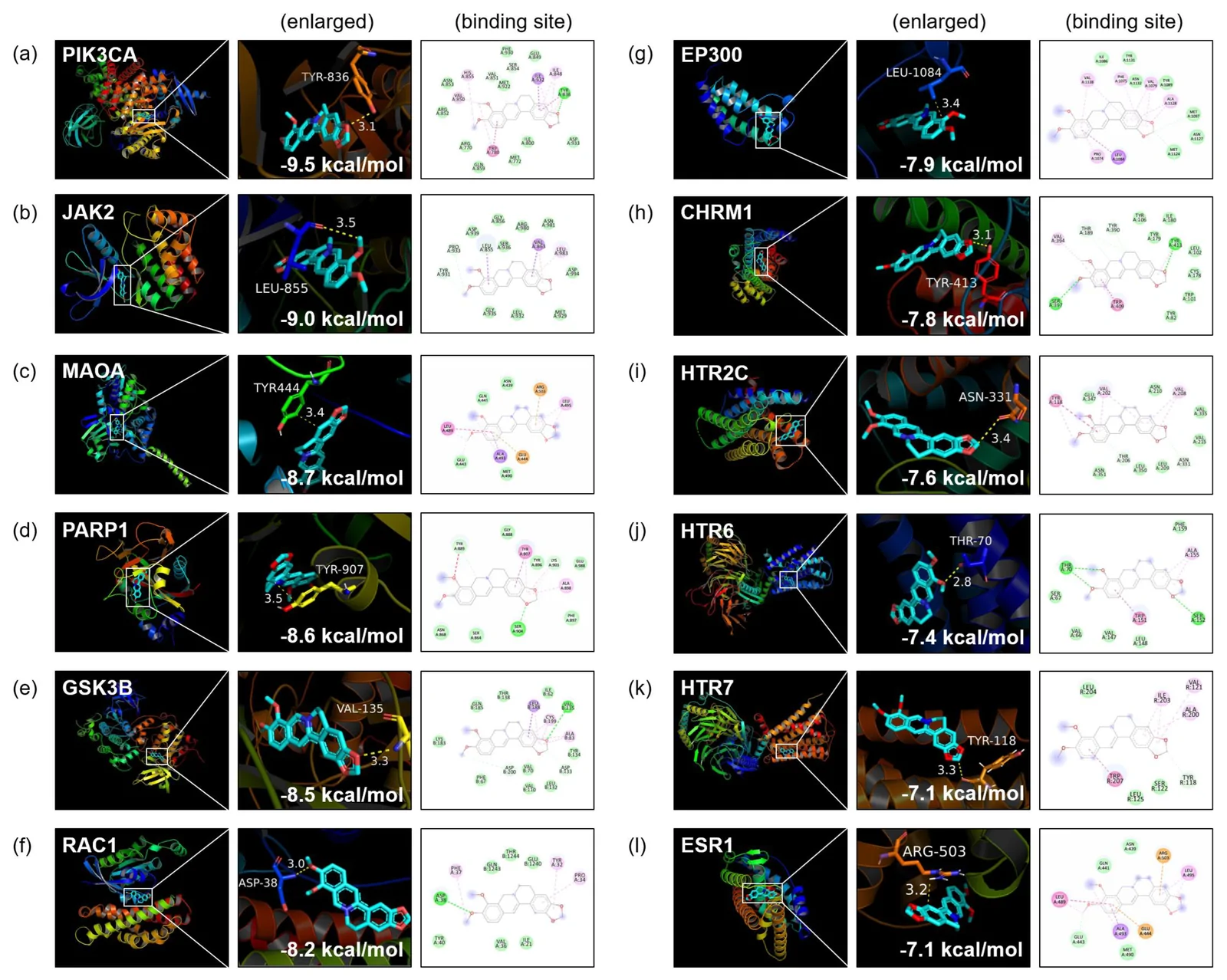
Molecular docking analysis of berberine with key target proteins associated with obesity-, dyslipidemia-, and diabetes-related metabolic dysfunction. Three-dimensional docking structures, enlarged binding pocket views, and two-dimensional interaction diagrams are shown for PIK3CA (**a**), JAK2 (**b**), MAOA (**c**), PARP1 (**d**), GSK3B (**e**), RAC1 (**f**), EP300 (**g**), CHRM1 (**h**), HTR2C (**i**), HTR6 (**j**), HTR7 (**k**), and ESR1 (**l**). Docking scores are indicated in kcal/mol within the enlarged binding pocket panels.

The molecular docking analysis generated predicted docking scores for berberine against all selected target proteins. Among the analyzed targets, the lowest docking scores were observed for PIK3CA (−9.5 kcal/mol) and JAK2 (−9.0 kcal/mol), followed by MAOA (−8.7 kcal/mol), PARP1 (−8.6 kcal/mol), GSK3B (−8.5 kcal/mol), RAC1 (−8.2 kcal/mol), EP300 (−7.9 kcal/mol), CHRM1 (−7.8 kcal/mol), HTR2C (−7.6 kcal/mol), HTR6 (−7.4 kcal/mol), and HTR7 and ESR1 (−7.1 kcal/mol each). The detailed docking scores are summarized in Table 6 and Figure 9.

## 3. Discussion

Berberine is a major bioactive alkaloid found in *Coptidis chinensis* and several other medicinal plants widely used in East Asian medicine. It has been extensively investigated for its metabolic effects, with previous studies reporting modest but significant improvements in obesity-related parameters and beneficial effects on glucose and lipid metabolism [40,41,42]. Coptidis Rhizoma, however, contains several other bioactive alkaloids, including coptisine, epiberberine, jatrorrhizine, and palmatine. Berberine was therefore selected not as the sole active constituent of Coptidis Rhizoma, but as a representative major alkaloid with the most extensively characterized clinical and mechanistic evidence relevant to obesity, diabetes, and dyslipidemia. Its comparatively well-established compound–target information also enabled cross-disease network pharmacology and molecular docking analyses.

The present meta-analysis demonstrated that Coptidis-containing formulations were associated with significant improvements in obesity parameters (BMI, BW, and WC), glycemic indices (FPG, 2hPG, HbA1c, and HOMA-IR), and lipid profiles (TC, TG, LDL-C, and HDL-C). While these findings align broadly with isolated reports on berberine [40,41,42], the clinical interventions evaluated here were multicomponent herbal formulations rather than purified berberine, and most were administered as add-on regimens to standard conventional therapies. Consequently, the observed clinical benefits cannot be attributed solely to berberine and likely reflect synergistic contributions from multiple co-occurring botanical constituents. Furthermore, substantial between-study heterogeneity was observed across several pooled outcomes, reflecting differences in herbal composition, dosage regimens, treatment duration, and baseline metabolic status. These findings support a multidomain metabolic benefit of Coptidis-containing formulations rather than a uniform or formulation-independent class effect.

Importantly, the certainty of the cumulative clinical evidence must be interpreted with prudent caution. In our GRADE assessment (Table A2), the certainty of evidence for most anthropometric, glycemic, and lipid outcomes was graded as low (and very low for waist circumference), with only HOMA-IR achieving moderate certainty. This rating was primarily driven by serious risk of bias—notably the widespread absence of double-blinding and placebo controls—alongside statistical heterogeneity. This methodological limitation often reflects the inherent challenge unique to botanical medicine trials, where the distinctive taste, aroma, and appearance of multi-herb decoctions hinder the fabrication of indistinguishable placebos. While these trials largely represent pragmatic, real-world add-on treatment settings, the lack of blinding inevitably introduces potential performance and detection biases. Thus, these pooled estimates should be viewed as promising therapeutic signals rather than definitive proof of standalone efficacy.

Our cross-disease target mapping identified 94, 95, and 54 overlapping targets for obesity, diabetes, and dyslipidemia, respectively, with MAOA, PIK3CA, PARP1, EP300, and ESR1 emerging as core hub genes across all three disease networks. Functional enrichment analysis further confirmed that key regulatory pathways were shared across the metabolic triad (Figure 8). The consistent enrichment of cAMP, PI3K–Akt, and calcium signaling cascades aligns with their established roles in insulin transduction, lipid metabolism, and inflammatory modulation [43,44,45,46,47,48,49]. PIK3CA was particularly prominent as both a central PPI hub and an essential kinase in the PI3K–Akt pathway. In addition, neuroactive ligand–receptor pathways (including HTR2C, HTR6, HTR7, CHRM1, and MAOA) highlight potential neuroendocrine involvement in appetite regulation and energy expenditure [50,51,52,53,54,55,56], while PARP1, EP300, and ESR1 link berberine targets to transcriptional regulation and metabolic stress responses [57,58,59]. Nevertheless, pathway enrichment reflects statistical associations within annotated databases and does not establish that these cascades were directly modulated in trial participants.

Molecular docking simulations subsequently confirmed favorable structural binding compatibility between berberine and prioritized hub targets, notably HTR6, PIK3CA, JAK2, MAOA, and PARP1. While these computational findings support the structural plausibility of selected compound–target engagements, docking simulations cannot establish physiological binding occupancy, downstream signaling kinetics, or causal efficacy in vivo. Accordingly, the network pharmacology and docking analyses should be interpreted as mechanistic hypotheses that contextualize, rather than validate, the observed clinical trial outcomes.

Taken together, this integrated approach highlights the unique translational value of the present study. While several systematic reviews have previously evaluated Coptidis Rhizoma or its principal alkaloid, berberine, most existing clinical evidence has focused on isolated conditions—such as type 2 diabetes [60], dyslipidemia [14], or obesity parameters [15]. However, in real-world clinical practice, these conditions frequently progress as an interconnected metabolic cluster. Assessing each condition separately overlooks the multi-systemic metabolic regulation of herbal formulations. Furthermore, while network pharmacology has previously been applied to investigate berberine in single-disease contexts, conventional in silico studies often operate in isolation without direct integration into synthesized clinical trial evidence. To overcome these limitations, the present study establishes a translational cross-disease framework. Clinically, our meta-analysis simultaneously evaluates the aggregated therapeutic signals of Coptidis-containing formulations across the metabolic triad. Mechanistically, rather than predicting generic compound–target interactions, we directly cross-referenced the shared clinical outcomes against berberine’s multi-target convergence across these comorbid conditions, effectively bridging macro-level clinical phenotypes with micro-level molecular mechanisms.

Several limitations warrant consideration. The relative contributions of berberine versus other major alkaloids (e.g., coptisine, epiberberine, jatrorrhizine, and palmatine) and their potential pharmacokinetic interactions remain unresolved. It is also uncertain whether predicted target affinities are achieved at physiologically relevant systemic concentrations. Furthermore, the included clinical trials were conducted exclusively in China, which may restrict generalizability to diverse populations and clinical environments. Methodological heterogeneity among trials, the static nature of public network databases, and the predictive assumptions of docking simulations further limit definitive causal inferences. Additionally, the molecular docking analysis did not include reference standard ligands or native co-crystallized ligand redocking for comparative benchmark validation, and these computational scores should strictly be interpreted as predictive binding plausibilities rather than experimental proof of target engagement.

Future research should decouple clinical and mechanistic validation. Multicenter, double-blind RCTs utilizing chemically standardized and batch-controlled Coptidis formulations are required to confirm the reproducibility and effect size across diverse ethnic cohorts. Mechanistically, multi-component analyses integrating the broader alkaloid profile alongside targeted experimental validations of prioritized kinase and receptor targets are warranted to delineate the holistic pharmacology of Coptidis Rhizoma.

## 4. Materials and Methods

### 4.1. Integrated Research of Meta-Analysis and In Silico Study

This study integrated a meta-analysis with in silico modeling to comprehensively evaluate the effects of Coptidis-containing herbal formulations on obesity, diabetes and dyslipidemia. First, RCTs investigating the efficacy of herbal prescriptions containing Coptidis Rhizoma for obesity, with or without metabolic comorbidities, were systematically identified to ensure an adequate evidence base. Subsequently, quantitative synthesis was performed using Review Manager (v. 5.4.1), focusing on key metabolic outcomes, including BMI, glucose-related parameters, and lipid profiles. In parallel, in silico analyses were conducted to explore the molecular mechanisms of berberine, a representative bioactive alkaloid of Coptidis Rhizoma. Among the constituents, berberine was selected as the representative compound based on its pharmacokinetic profiles and extensive literature evidence.

### 4.2. Search Strategy

The systematic review and meta-analysis were conducted in accordance with the PRISMA 2020 statement. All investigators were involved in the literature search, study selection, and data extraction processes. Eligible studies evaluating the efficacy and safety of Coptidis Rhizoma (Huanglian)-containing interventions for obesity, diabetes, and dyslipidemia were systematically identified through electronic database searches. Two major databases, PubMed and the Chinese National Knowledge Infrastructure (CNKI), were queried to retrieve relevant publications from inception through March 2026.

### 4.3. Inclusion and Exclusion Criteria

#### 4.3.1. Study Design

Only RCTs investigating the efficacy and safety of Coptidis Rhizoma (Huanglian, 黃連)-containing interventions for obesity with or without metabolic comorbidities were considered eligible, without restrictions on language or publication date. Studies were excluded based on the following criteria: (1) non-randomized study designs; (2) lack of relevance to obesity or dyslipidemia; (3) absence of Coptidis Rhizoma as a principal component of the intervention; (4) use of non-oral routes of administration; (5) publication types such as case reports, reviews, or conference abstracts; (6) preclinical or in silico studies including animal, in vitro experiments, or stand-alone network pharmacology without clinical trial data; and (7) failure to report lipid-related outcomes or adiposity-related parameters as primary endpoints. Literature screening was conducted on studies published up to March 2026.

#### 4.3.2. Participant Characteristics

No restrictions were applied regarding participant characteristics such as age, sex, or ethnicity. Eligible studies were required to include individuals presenting with obesity and/or dyslipidemia and diabetes as the primary clinical conditions, defined according to established diagnostic criteria. To maintain a focused assessment of metabolic outcomes, studies in which these conditions were secondary to other major diseases were generally excluded. Specifically, studies involving participants with diabetes mellitus were retained for additional analyses, given the close metabolic interplay between obesity, dyslipidemia, and diabetes.

#### 4.3.3. Types of Interventions

RCTs evaluating Coptidis-containing formulations as the primary intervention in comparison with a control group were included. Eligible interventions were limited to orally administered decoction-based formulations containing Coptidis Rhizoma, with no restrictions on dosage, composition, or treatment duration. Studies employing non-oral routes of administration were excluded. Both monotherapy and adjunctive therapies (Coptidis formulations combined with conventional pharmacotherapy versus conventional pharmacotherapy alone) were eligible, provided that Coptidis-containing formulations were mainly under investigation.

#### 4.3.4. Outcome Measures

The primary outcomes included obesity-related indices such as BMI, body weight and waist circumference. Glycemic parameters, including FPG, 2hPG, HbA1c, and insulin resistance indices such as the HOMA-IR, were also assessed. In addition, lipid profile parameters, including TC, TG, HDL-C, and LDL-C, were also extracted to provide a comprehensive evaluation of metabolic effects.

### 4.4. Methodological Quality Assessment

The methodological quality of the included studies was independently evaluated by multiple reviewers using the Revman program. This tool assesses potential bias across five domains: the randomization process, deviations from intended interventions, missing outcome data, measurement of outcomes, and selective reporting of results. Each study was categorized as having a low risk of bias, unclear risk, or a high risk of bias according to established criteria. Any discrepancies between reviewers were resolved through discussion and consensus, with arbitration by the corresponding author when necessary.

### 4.5. Assessment of Risk of Bias and Quality of Evidence

The certainty of evidence for each major meta-analyzed outcome was evaluated using the GRADE approach. Evidence derived from RCTs was initially considered high certainty and was downgraded, where appropriate, according to five domains: risk of bias, inconsistency, indirectness, imprecision, and publication bias. The resulting certainty of evidence was classified as high, moderate, low, or very low. The outcome-specific certainty ratings and the principal reasons for downgrading are explicitly reported in Section 2.2 of the Results.

### 4.6. Statistical Analysis

All statistical analyses were conducted using Review Manager. Included trials were stratified according to the characteristics of the intervention and comparator groups. For continuous outcomes, pooled effect sizes were calculated with corresponding 95% CI, and SMD were applied when outcome measures were reported using different units or scales. The analyzed endpoints comprised obesity, glycemic and lipid parameters—BMI, weight, waist circumference, FPG, 2hPG, HbA1c, HOMA-IR, TC, TG, HDL-C, and LDL-C. Between-study heterogeneity was evaluated using the Cochran’s Q test and the I^2^ statistic, where an I^2^ > 50% indicated substantial heterogeneity. To account for potential clinical variations across the included trials—such as differences in herbal compositions, dosages, and treatment durations—a random-effects model was systematically applied for all pooled analyses. Potential small-study effects and publication bias were further explored using contour-enhanced funnel plots.

### 4.7. ADME Evaluation of Berberine

The ADME properties of berberine were evaluated using the Traditional Chinese Medicine Systems Pharmacology Database and Analysis Platform (TCMSP) to assess its pharmacokinetic suitability as a representative compound of Coptidis Rhizoma. The assessed pharmacokinetic parameters included molecular weight, OB, DL, Caco-2 permeability, BBB permeability, and half-life. Commonly used screening criteria, including OB ≥ 30%, Caco-2 permeability > −0.4, and DL ≥ 0.18, were applied to evaluate whether berberine exhibited acceptable pharmacokinetic properties for subsequent in silico analyses.

### 4.8. Prediction of Metabolic Regulatory Mechanisms of Berberine Based on Network Pharmacology

A network pharmacology analysis was performed to explore the potential molecular mechanisms of berberine in obesity-, diabetes-, and dyslipidemia-related metabolic dysfunction. Potential target genes of berberine were predicted using the SwissTargetPrediction platform. Disease-related target genes were retrieved from the GeneCards database on 8 June 2026, using the keywords “obesity,” “dyslipidemia,” and “diabetes”.

Using Venny 2.1, overlapping targets between berberine-related targets and each disease-related target set were identified. The common targets between berberine and obesity, diabetes, and dyslipidemia were used for subsequent PPI network construction and functional enrichment analyses.

PPI networks were constructed using the STRING database. For the initial PPI network construction and hub gene identification, the minimum required interaction score was set to a high-confidence threshold of 0.7. The resulting PPI networks were imported into Cytoscape version 3.10.4 for visualization and topological analysis. Network centrality indices, including degree, betweenness centrality, and closeness centrality, were calculated, and hub genes were selected primarily based on degree centrality. To visualize the interactions among the selected hub genes, the top 12–15 hub genes for each disease category were resubmitted to STRING using a medium-confidence interaction score threshold of 0.4. The resulting hub-gene subnetworks were transferred to Cytoscape and visualized using a circular layout.

To characterize the biological functions of the common targets, GO functional enrichment analysis was performed using DAVID Bioinformatics Resources. GO analysis included three categories: BP, CC, and MF. For each disease category, the top five GO terms in each BP, CC, and MF category were selected based on the number of involved target proteins. KEGG pathway enrichment analysis was also performed using DAVID, and the top enriched pathways were selected primarily based on target count. GO and KEGG enrichment results were visualized as bubble plots using the ggplot2 package in RStudio (v. 2026.04.0), with the x-axis representing the number of proteins, bubble size indicating target count, and bubble color representing the *p*-value.

### 4.9. Molecular Docking Analysis

Based on the network pharmacology, PPI hub-gene analysis, and KEGG pathway enrichment results, molecular docking analysis was performed to further investigate the potential interactions between berberine and representative target proteins associated with obesity-, diabetes-, and dyslipidemia-related metabolic dysfunction. Target proteins were selected using a combined strategy. First, common hub proteins identified across the obesity-, diabetes-, and dyslipidemia-related PPI subnetworks were included to represent central molecular nodes shared by the three metabolic disease categories. Second, additional proteins were selected based on their recurrent occurrence across highly enriched KEGG pathways and their biological relevance to lipid metabolism, inflammatory responses, PI3K-Akt signaling, insulin signaling, and neuroactive ligand-related signaling.

Accordingly, the final docking targets consisted of PPI-derived common hub proteins, namely MAOA, PIK3CA, PARP1, EP300, and ESR1, and KEGG pathway-associated representative proteins, namely RAC1, CHRM1, HTR6, HTR7, HTR2C, GSK3B, and JAK2. PIK3CA was included as both a common PPI hub protein and a pathway-relevant target associated with PI3K-Akt signaling.

The three-dimensional structure of berberine was obtained from the PubChem database. The three-dimensional structures of the selected target proteins, including PIK3CA (PDB ID: 8EXL), RAC1 (PDB ID: 5O33), CHRM1 (PDB ID: 3BKB), HTR6 (PDB ID: 4PNR), HTR7 (PDB ID: 4KAK), HTR2C (PDB ID: 5Q1S), GSK3B (PDB ID: 3DDW), JAK2 (PDB ID: 7Q7L), MAOA (PDB ID: 2Z5X), PARP1 (PDB ID: 6NRG), EP300 (PDB ID: 5LPM), and ESR1 (PDB ID: 1X7R), were downloaded from the RCSB Protein Data Bank database.

The criteria for selecting PDB entries were as follows: protein structures were restricted to Homo sapiens origin when available; structures determined by X-ray diffraction were preferentially selected; and entries with resolutions close to or below 2.0 Å and clearly identifiable ligand-binding domains were prioritized. When multiple PDB structures were available for the same target, the structure with better resolution, appropriate ligand-binding information, and suitability for docking preparation was selected.

Prior to molecular docking, the downloaded protein structures were prepared in PyMOL (v. 3.0.0) by removing water molecules and heteroatoms, including co-crystallized ligands, and imported into PyRx. The berberine ligand structure was prepared and energy-minimized using Open Babel.

Molecular docking was conducted via AutoDock (v. 1.5.6) Vina in PyRx. For each target, search spaces were defined using grid boxes centered on the established active binding sites, with specific dimensions, center coordinates, and generated pose numbers detailed in Table A3. The exhaustiveness parameter was set to 8. Up to nine binding poses were generated per target, and the conformation with the lowest docking score (mode 0, in kcal/mol) was selected as the representative pose. While redocking validation of native co-crystallized ligands was not performed, search spaces were mapped directly to structurally characterized catalytic pockets in the respective PDB structures.

Docking poses and ligand–protein interaction profiles (including hydrogen bonds and hydrophobic interactions) were visualized using PyMOL and BIOVIA Discovery Studio Visualizer.

## 5. Conclusions

In conclusion, this integrated study indicates that Coptidis-containing formulations may improve multiple metabolic outcomes related to obesity, dyslipidemia, and glucose metabolism. The meta-analysis provides clinical evidence of these effects, while the network pharmacology and molecular docking analyses offer a complementary mechanistic framework by identifying berberine-associated targets and pathways involved in lipid metabolism, insulin signaling, inflammation, and kinase-mediated signaling. Thus, the in silico findings provide biological plausibility for the metabolic effects observed in the meta-analysis rather than direct mechanistic validation. Given the heterogeneity among the included clinical studies and the predictive nature of the computational analyses, well-designed multicenter randomized controlled trials and experimental studies are required to confirm the clinical efficacy and underlying mechanisms of Coptidis Rhizoma and berberine.

## Figures and Tables

**Table 1:** Characteristics of each experiment.

First Author	Participants	Number of Cases/Age, Years		Intervention Methods		Observation Period	Outcome Indicators
		Trial Group	Control Group	Trial Group	Control Group		
Li, 2026 [21]	Obese, diabetic	40/52.23 ± 4.49	40/53.67 ± 4.82	Huanglian Heye Formula with dulaglutide	dulaglutide	12 weeks	BMI, FPG, 2hPG, HbA1c, HOMA-IR
Zhang, 2025 [22]	obese, diabetic	47/54.25 ± 9.17	47/53.48 ± 9.56	Huanglian Heye Formula with insulin	Huanglian Heye Formula with insulin	12 weeks	WHR, BMI, TC, TG, LDL-C, HbA1c, 2hPG, FPG, VASPIN, HOMA-B,
Han, 2025 [23]	obese, hypertensive	34/55 ± 8	34/54 ± 7	Huanglian Wendan decoction with conventional treatment	conventional treatment	8 weeks	BMI, WC, TG, TC, LDL-C, HDL-C, homocysteine. sBP, dBP
Jin, 2025 [24]	obese, diabetic, dyslipidemia	30/58.76 ± 3.92	30/58.48 ± 3.89	Huanglian Jiangtang Pill with metformin	metformin	4 weeks	TG, TC, LDL-C, HDL-C, FPG, 2hPG, HbA1c
Liang, 2022 [25]	obese, diabetic	50/53.18 ± 4.32	53.34 ± 4.41	Huanglian Jiedu decoction with metformin and conventional treatment	metformin with conventional treatment	12 weeks	TC, TG, HDL-C, LDL-C, FPG, 2hPG, HbA1c, FINS, HOMA-IR, HOMA-B, Cortisol
Fu, 2022 [26]	obese, diabetic	98/51.9 ± 4.3	98/52.2 ± 4.7	Huanglian Jiedu decoction with liraglutide	liraglutide	8 weeks	BMI, BW, BFR, FPG, 2hPG, HbA1c, FINS, HOMA-IR, HOMA-B
Han, 2022 [27]	obese, diabetic	60/ 53.68 ± 10.11	60/ 54.12 ± 9.89	Huanglian Heye formula with metformin	metformin	12 weeks	BMI, WC, HC, WHR/FPG, 2hPG, HbA1c, HOMA-IR, RHI
Xiao, 2022 [28]	obese, diabetic	34/unknown	31/unknown	Huanglian Wendan Decoction with lixisenatide and metformin	lixisenatide and metformin	12 weeks	BMI, WC, BW, TC, TG, HDL-C, LDL-C, FPG, 1hPG, 2hPG, HbA1c, FINS, 1hINS, 2hINS, HOMA-IR
Ye, 2021 [29]	obese, diabetic	68/52.14 ± 13.91	68/51.45 ± 13.46	Huanglian Qingre Tongluo Recipe with Liraglutide	liraglutide	8 weeks	BMI, WC, FPG, 2hPG, HOMA-IR, SOD, MDA, T-AOC, Visceral fat volume, Subcutaneous fat volume
Chen, 2021 [30]	obese, diabetic	50/54.37 ± 2.56	49/54.41 ± 2.24	Modified Huanglian Jiedu Decoction with metformin	metformin	12 weeks	TC, TG, HDL-C, LDL-C, FPG, 2hPG, HbA1c, FINS, HOMA-IR
An, 2021 [31]	obese, diabetic, dyslipidemia	36/unknown	38/unknown	Huanglian turbidity resolving prescription with metformin	metformin	16 weeks	BMI
Wei, 2021 [32]	obese, diabetic	30/53.68 ± 5.02	30/55.05 ± 4.82	Huanglian Jiedu Decoction with conventional treatment	conventional treatment	12 weeks	BMI, FPG, 2hPG, HbA1c, FINS, C-P, HOMA-B, APN
Yin 2020 [33]	obese, diabetic	50/58.58 ± 3.28	50/58.69 ± 3.33	Huanglian Jiedu Decoction with conventional treatment	conventional treatment	3 weeks	BMI, FPG, 2hPG, HbA1c
Kong, 2019 [34]	obese, diabetic	30/50.72 ± 10.13	30/54.54 ± 8.22	Huanglian Wendan decoction with conventional treatment	metformin and conventional treatment	12 weeks	BMI, WC, BW, TG, TC, LDL-C, HDL-C, FPG, 2hPG
Zhang, 2015 [35]	obese, diabetic	30/56.71 ± 8.50	30/56.67 ± 7.74	Huanglian Jiedu decoction	metformin	12 weeks	TC, TG, LDL-C, HDL-C, FPG, 2hPG, HbA1c, CPRT, IRT, HOMA-IR, HOMA-IS
Tu, 2015 [36]	obese, diabetic	79/52.6 ± 16.3	79/53.5 ± 16.6	Huanglian Jiedu decoction	metformin	12 weeks	BMI, weight loss efficacy. FPG, 2hPG, HbA1c
Ji, 2013 [37]	obese, diabetic	30/43 ± 12	30/46 ± 14	Huanglian Jiedu decoction with insulin	metformin and insulin	12 weeks	BMI, TC, TG, LDL-C, HDL-C, FPG, 2hPG, HbA1c, HOMA-IR, HOMA-IS, time to glycemic control, insulin dose, hs-CRP, IL-6
Li, 2013 [38]	obese, diabetic	30/58.90 ± 12.6	30/60.13 ± 11.5	Huanglian Jiedu decoction	metformin	12 weeks	BMI, TC, TG, LDL-C, HDL-C, FPG, 2hPG, HbA1c, OGTT (0,1,2,3H), CPRT, IRT, HOMA-IR, HOMA-IS, HBCI, hs-CRP, IL-6, ET-1, TXB2, PGF1A, NO
Wang, 2011 [39]	obese, diabetic	30/42 ± 16	30/40 ± 15	Huanglian Jiedu decoction granules with insulin and metformin	insulin and metformin	6 months	BMI, FPG, 2hPG, HbA1c, time to glycemic control, insulin dose, OGTT (0,1,2,3H), CPRT, IRT, HOMA-IR

1hINS, 1 h insulin; 1hPG, 1 h postload glucose; 2hINS, 2 h insulin; 2hPG, 2 h postload glucose; APN, adiponectin; BFR, body fat rate; BMI, body mass index; BW, body weight; C-P, C-peptide; CPRT, C-peptide release test; dBP, diastolic blood pressure; ET-1, endothelin-1; FINS, fasting insulin; FPG, fasting plasma glucose; HbA1c, hemoglobin A1c; HBCI, homeostasis model assessment beta-cell function index; HC, hip circumference; HDL-C, high-density lipoprotein cholesterol; HOMA-B, homeostasis model assessment of beta-cell function; HOMA-IR, homeostasis model assessment of insulin resistance; HOMA-IS, homeostasis model assessment of insulin sensitivity; hs-CRP, high-sensitivity C-reactive protein; IL-6, interleukin-6; INS, insulin; IRT, insulin release test; LDL-C, low-density lipoprotein cholesterol; MDA, malondialdehyde; NO, nitric oxide; OGTT, oral glucose tolerance test; PGF1A, prostaglandin F1 alpha; RHI, reactive hyperemia index; sBP, systolic blood pressure; SOD, superoxide dismutase; T-AOC, total antioxidant capacity; TC, total cholesterol; TG, triglycerides; TXB2, thromboxane B2; WC, waist circumference; WHR, waist-to-hip ratio.

**Table 2:** Meta-analysis summary of body mass index.

	Std. Mean Difference	Heterogeneity
Coptidis + antidiabetic vs. antidiabetic (n = 6)	−0.87 [−1.49, −0.25]	*p* < 0.00001, I^2^ = 91%
Coptidis + GLP-1 RA vs. GLP-1 RA (n = 3)	−0.89 [−1.19, −0.60]	*p* = 0.15, I^2^ = 46%
Coptidis vs. metformin (n = 3)	−0.10 [−0.33, 0.14]	*p* = 0.88, I^2^ = 0%
Coptidis + conventional vs. conventional (n = 4)	−0.82 [−1.20, −0.45]	*p* = 0.08, I^2^ = 56%
Total	−0.72 [−1.00, −0.44]	*p* < 0.00001, I^2^ = 85%

**Table 3:** Meta-analysis summary of glycated hemoglobin.

	Std. Mean Difference	Heterogeneity
Coptidis + antidiabetic vs. antidiabetic (n = 6)	−0.93 [−1.50, −0.36]	*p* < 0.00001, I^2^ = 89%
Coptidis + GLP-1 RA vs. GLP-1 RA (n = 3)	−0.34 [−0.56, −0.13]	*p* = 0.36, I^2^ = 2%
Coptidis vs. metformin (n = 3)	−0.69 [−1.45, −0.07]	*p* = 0.0008, I^2^ = 88%
Coptidis + conventional vs. conventional (n = 2)	−1.09 [−2.05, −0.13]	*p* = 0.0001, I^2^ = 86%
Total	−0.77 [−1.07, −0.47]	*p* < 0.00001, I^2^ = 85%

**Table 4:** Meta-analysis summary of low-density lipoprotein cholesterol.

	Std. Mean Difference	Heterogeneity
Coptidis + antidiabetic vs. antidiabetic (n = 5)	−1.20 [−2.02, −0.37]	*p* < 0.00001, I^2^ = 93%
Coptidis vs. metformin (n = 2)	−0.72 [−1.09, −0.35]	*p* = 0.51, I^2^ = 0%
Coptidis + conventional vs. conventional (n = 3)	−1.20 [−2.13, −0.26]	*p* < 0.0001, I^2^ = 90%
Total	−1.10 [−1.57, −0.62]	*p* < 0.00001, I^2^ = 89%

**Table 5:** ADME properties of berberine obtained from the TCMSP database.

Compound	MW	OB (%)	DL	Caco-2	BBB	HL
Berberine	336.39	36.86	0.78	1.24	0.57	6.57

MW, molecular weight; OB, oral bioavailability; DL, drug likeness; Caco-2, Caco-2 permeability; BBB, blood–brain barrier penetration; HL, half life.

**Table 6:** Predicted docking scores of berberine to common target proteins overlapping among obesity-, dyslipidemia-, and diabetes-related networks.

Target Protein	PDB ID	Docking Score (kcal/mol)
PIK3CA	8EXL	−9.5
JAK2	7Q7L	−9.0
MAOA	2Z5X	−8.7
PARP1	6NRG	−8.6
GSK3B	5F94	−8.5
RAC1	5O33	−8.2
EP300	5LPM	−7.9
CHRM1	9JEA	−7.8
HTR2C	9UGL	−7.6
HTR6	7YS6	−7.4
HTR7	7XTC	−7.1
ESR1	1X7R	−7.1

## Data Availability

Data are contained within the article.

## Abbreviations

The following abbreviations are used in this manuscript:

LDL-C	low-density lipoprotein cholesterol
TG	triglycerides
GLP1-RA	GLP-1 receptor agonists
RCTs	randomized controlled trials
CNKI	China National Knowledge Infrastructure
GRADE	Grading of Recommendations Assessment, Development and Evaluation
BMI	body mass index
BW	body weight
WC	waist circumference
HbA1c	glycated hemoglobin
FPG	fasting plasma glucose
2hPG	2—hour postprandial glucose
HOMA-IR	homeostatic model assessment of insulin resistance
TC	total cholesterol
TG	triglyceride
HDL-C	high-density lipoprotein cholesterol
SMD	standardized mean difference
ADME	absorption, distribution, metabolism, and excretion
OB	oral bioavailability
DL	drug-likeness
BBB	Blood–brain barrier
PPI	protein–protein interaction
GO	Gene Ontology
KEGG	Kyoto Encyclopedia of Genes and Genomes
BP	Biological Process
CC	Cellular Component
MF	Molecular Function
GPCR	G protein-coupled receptor
PI3K	Phosphoinositide 3-kinase
TLR	Toll-like receptor
NAFLD	non-alcoholic fatty liver disease
PIK3CA	Phosphatidylinositol-4,5-Bisphosphate 3-Kinase Catalytic Subunit Alpha
RAC1	Rac Family Small GTPase 1
CHRM1	Cholinergic Receptor Muscarinic 1
HTR6	5-Hydroxytryptamine Receptor 6
HTR7	5-Hydroxytryptamine Receptor 7
HTR2C	5-Hydroxytryptamine Receptor 2C
GSK3B	Glycogen Synthase Kinase 3 Beta
JAK2	Janus Kinase 2
MAOA	Monoamine Oxidase A
PARP1	Poly(ADP-Ribose) Polymerase 1
EP300	E1A Binding Protein P300
ESR1	Estrogen Receptor 1
TCMSP	Traditional Chinese Medicine Systems Pharmacology Database and Analysis Platform

## Appendix A

### Appendix A.1. Primary Source Bibliographic Records and Direct Access Information

**Table A1:** Summary of document types, issuing sources, and direct overseas access repositories for all included RCTs.

Study ID	Document Type	Source Journal/Issuing University	Direct Access (CNKI/Repository)
Li, 2026 [21]	Journal Article	Acta Chinese Medicine and Pharmacology	[Direct CNKI Access]
Zhang, 2025 [22]	Journal Article	Journal of Guangzhou University of Traditional Chinese Medicine	[Direct CNKI Access]
Han, 2025 [23]	Journal Article	China Medicine	[Direct CNKI Access]
Jin, 2025 [24]	Journal Article	Diabetes New World	[Direct CNKI Access]
Liang, 2022 [25]	Journal Article	China’s Naturopathy	[Direct CNKI Access]
Fu, 2022 [26]	Journal Article	Chinese Archives of Traditional Chinese Medicine	[Direct CNKI Access]
Han, 2022 [27]	Journal Article	Journal of Beijing University of Traditional Chinese Medicine	[Direct CNKI Access]
Xiao, 2022 [28]	Master’s Thesis	Chengdu University of Traditional Chinese Medicine	[Direct CNKI Access]
Ye, 2021 [29]	Journal Article	Journal of Sichuan of Traditional Chinese Medicine	[Direct CNKI Access]
Chen, 2021 [30]	Journal Article	Zhejiang Journal of Traditional Chinese Medicine	[Direct CNKI Access]
An, 2021 [31]	Journal Article	Western Journal of Traditional Chinese Medicine	[Direct CNKI Access]
Wei, 2021 [32]	Journal Article	World Journal of Complex Medicine	[Direct CNKI Access]
Yin 2020 [33]	Journal Article	Diabetes New World	[Direct CNKI Access]
Kong, 2019 [34]	Master’s Thesis	Zhejiang Chinese Medical University	[Direct CNKI Access]
Zhang, 2015 [35]	Master’s Thesis	Shandong University of Traditional Chinese Medicine	[Direct CNKI Access]
Tu, 2015 [36]	Journal Article	China Journal of Chinese Medicine	[Direct CNKI Access]
Ji, 2013 [37]	Master’s Thesis	Shandong University of Traditional Chinese Medicine	[Direct CNKI Access]
Li, 2013 [38]	Master’s Thesis	Shandong University of Traditional Chinese Medicine	[Direct CNKI Access]
Wang, 2011 [39]	Master’s Thesis	Shandong University of Traditional Chinese Medicine	[Direct CNKI Access]

### Appendix A.2. Certainty of Evidence Assessment Using the GRADE Approach

**Table A2:** GRADE Evidence Profile and Summary of Findings for Meta-Analyzed Outcomes (N = 19).

Outcome	Pooled Effect Size (95% CI)	Studies (N)	Total Patients	Certainty (GRADE)	Main Downgrading Reasons
BMI (kg/m^2^)	SMD: −0.72 [−1.00, −0.44]	16	1479	Low (⨁⨁◯◯)	Risk of bias ^a^, Inconsistency (I^2^ = 85%) ^b^
BW (kg)	SMD: −0.31 [−0.53, −0.09]	3	321	Low (⨁⨁◯◯)	Risk of bias ^a^, Imprecision ^c^
WC (cm)	SMD: −0.78 [−1.33, −0.24]	5	449	Low (⨁⨁◯◯)	Risk of bias ^a^, Inconsistency (I^2^ = 86%) ^b^
FPG (mmol/L)	SMD: −0.93 [−1.23, −0.64]	15	1469	Low (⨁⨁◯◯)	Risk of bias ^a^, Inconsistency (I^2^ = 86%) ^b^
2hPG (mmol/L)	SMD: −0.92 [−1.18, −0.66]	16	1529	Low (⨁⨁◯◯)	Risk of bias ^a^, Inconsistency (I^2^ = 83%) ^b^
HbA1c (%)	SMD: −0.77 [−1.07, −0.47]	14	1293	Low (⨁⨁◯◯)	Risk of bias ^a^, Inconsistency (I^2^ = 85%) ^b^
HOMA-IR	SMD: −0.88 [−1.05, −0.70]	12	1146	Moderate (⨁⨁⨁◯)	Risk of bias ^a^, (I^2^ = 49%, consistent)
TC (mmol/L)	SMD: −0.73 [−1.09, −0.37]	11	839	Low (⨁⨁◯◯)	Risk of bias ^a^, Inconsistency (I^2^ = 84%) ^b^
TG (mmol/L)	SMD: −1.00 [−1.42, −0.58]	11	839	Low (⨁⨁◯◯)	Risk of bias ^a^, Inconsistency (I^2^ = 88%) ^b^
LDL-C (mmol/L)	SMD: −1.10 [−1.57, −0.62]	10	763	Low (⨁⨁◯◯)	Risk of bias ^a^, Inconsistency (I^2^ = 89%) ^b^
HDL-C (mmol/L)	SMD: +0.93 [+0.69, +1.17]	9	669	Low (⨁⨁◯◯)	Risk of bias ^a^, Inconsistency (I^2^ = 56%) ^b^

BMI, body mass index; BW, body weight; WC, waist circumference; FPG, fasting plasma glucose; 2hPG, 2 h postprandial glucose; HbA1c, glycated hemoglobin; HOMA-IR, homeostatic model assessment of insulin resistance; TC, total cholesterol; TG, triglycerides; LDL-C, low-density lipoprotein cholesterol; HDL-C, high-density lipoprotein cholesterol; CI, confidence interval; GRADE, Grading of Recommendations Assessment, Development, and Evaluation. *Certainty of evidence symbols according to* GRADE: ⨁⨁⨁⨁ High certainty; ⨁⨁⨁◯ Moderate certainty; ⨁⨁◯◯ Low certainty; ⨁◯◯◯ Very low certainty. ^a^ Risk of bias: Downgraded by 1 level due to lack of participant/personnel blinding (performance bias) and unclear allocation concealment across the included trials. ^b^ Inconsistency: Downgraded by 1–2 levels due to substantial between-study statistical heterogeneity (I^2^ > 50%). ^c^ ImprecisiDowngraded by 1 level due to small cumulative sample size and wide confidence intervals.

### Appendix A.3. Molecular Docking Simulation Parameters and Search Space Coordinates

**Table A3:** Grid-box coordinates, dimensions, and number of generated poses for molecular docking of berberine with selected target proteins.

Target	PDB ID	Center X	Center Y	Center Z	Dimension X (Å)	Dimension Y (Å)	Dimension Z (Å)	No. of Poses
PIK3CA	8EXL	−0.3086	10.1057	20.7670	81.8637	93.5050	100.6084	8
JAK2	7Q7L	28.7362	19.7084	70.5972	54.0601	54.2112	45.8247	9
MAOA	2Z5X	7.1152	−0.5744	22.7862	72.6205	68.2371	72.9306	9
PARP1	6NRG	25.2484	−3.9853	18.7393	57.7671	42.8526	41.8596	9
GSK3B	5F94	−17.413	−0.5583	21.0580	67.7897	69.4755	97.0983	9
RAC1	5O33	−8.0240	−15.1997	17.3795	56.1131	49.7749	66.8161	9
EP300	5LPM	34.6397	21.2456	8.1537	55.5968	53.3673	48.9853	9
CHRM1	9JEA	108.684	107.274	102.0418	46.6428	39.6901	89.9805	9
HTR2C	9UGL	127.1216	129.9692	160.2086	40.5133	54.7214	59.3805	9
HTR6	7YS6	139.913	135.382	139.3116	113.2117	118.2267	83.4662	9
HTR7	7XTC	114.883	94.2661	121.5435	87.5216	82.3700	108.8611	9
ESR1	1X7R	20.3813	27.6572	12.5400	47.6538	46.2520	56.9145	9

Note: Center coordinates ($X, Y, Z$) and dimensions (X, Y, Z) define the search space grid box used in AutoDock Vina simulations. Grid centers were mapped to the structurally characterized catalytic and active binding sites derived from the respective Protein Data Bank (PDB) structures. Å, Ångström; PDB, Protein Data Bank.

## Appendix B

### Appendix B.1. Body Weight

Two trials compared Coptidis-containing formulations plus GLP-1 RAs with GLP-1 RAs alone. A single trial compared Coptidis-containing formulations plus conventional treatment with conventional treatment alone. The corresponding results are summarized in Table A4 and Figure A1.

**Table A4:** Meta-analysis summary of body weight.

	Std. Mean Difference	Heterogeneity
Coptidis + GLP-1 RA vs. GLP-1 RA (n = 2)	−0.35 [−0.60, −0.11]	*p* = 0.42, I^2^ = 0%
Coptidis + conventional vs. conventional (n = 1)	−0.16 [−0.66, 0.35]	Not applicable
Total	−0.31 [−0.53, −0.09]	*p* = 0.57, I^2^ = 0%

### Appendix B.2. Waist Circumference

One trial evaluated the adjunctive use of Coptidis-containing formulations in combination with antidiabetic medications compared with antidiabetic medications alone. Two trials compared Coptidis-containing formulations plus GLP-1 RAs with GLP-1 RAs alone. Two trials compared Coptidis-containing formulations plus conventional treatment with conventional treatment alone. The corresponding results are summarized in Table A5 and Figure A2.

**Table A5:** Meta-analysis summary of waist circumference.

	Std. Mean Difference	Heterogeneity
Coptidis + antidiabetic vs. antidiabetic (n = 1)	−0.94 [−1.32, −0.56]	Not applicable
Coptidis + GLP-1 RA vs. GLP-1 RA (n = 2)	−0.36 [−1.67, 0.96]	*p* < 0.00001, I^2^ = 95%
Coptidis + conventional vs. conventional (n = 2)	−1.14 [−1.94, −0.34]	*p* = 0.03, I^2^ = 78%
Total	−0.78 [−1.33, −0.24]	*p* < 0.00001, I^2^ = 86%

### Appendix B.3. Fasting Plasma Glucose

Five trials evaluated the adjunctive use of Coptidis-containing formulations in combination with antidiabetic medications compared with antidiabetic medications alone. Four trials compared Coptidis-containing formulations plus GLP-1 RAs with GLP-1 RAs alone. Three trials assessed Coptidis-containing formulations combined with metformin versus metformin monotherapy, while three trials compared Coptidis-containing formulations plus conventional treatment with conventional treatment alone. The corresponding results are summarized in Table A6 and Figure A3.

**Table A6:** Meta-analysis summary off fasting plasma glucose.

	Std. Mean Difference	Heterogeneity
Coptidis + antidiabetic vs. antidiabetic (n = 5)	−1.07 [−1.79, −0.35]	*p* < 0.00001, I^2^ = 92%
Coptidis + GLP-1 RA vs. GLP-1 RA (n = 4)	−0.77 [−1.02, −0.53]	*p* = 0.18, I^2^ = 39%
Coptidis vs. metformin (n = 3)	−1.13 [−2.36, 0.10]	*p* < 0.00001, I^2^ = 95%
Coptidis + conventional vs. conventional (n = 3)	−0.90 [−1.33, −0.47]	*p* = 0.06, I^2^ = 64%
Total	−0.93 [−1.23, −0.64]	*p* = 0.29, I^2^ = 86%

### Appendix B.4. 2 h Postprandial Glucose

Six trials evaluated the adjunctive use of Coptidis-containing formulations in combination with antidiabetic medications compared with antidiabetic medications alone. Four trials compared Coptidis-containing formulations plus GLP-1 RAs with GLP-1 RAs alone. Three trials assessed Coptidis-containing formulations combined with metformin versus metformin monotherapy, while three trials compared Coptidis-containing formulations plus conventional treatment with conventional treatment alone. The corresponding results are summarized in Table A7 and Figure A4.

**Table A7:** Meta-analysis summary of 2 h postprandial glucose.

	Std. Mean Difference	Heterogeneity
Coptidis + antidiabetic vs. antidiabetic (n = 6)	−0.94 [−1.35, −0.52]	*p* = 0.0001, I^2^ = 80%
Coptidis + GLP-1 RA vs. GLP-1 RA (n = 4)	−0.77 [−0.96, −0.59]	*p* = 0.77, I^2^ = 0%
Coptidis vs. metformin (n = 3)	−0.68 [−1.39, 0.04]	*p* = 0.0007, I^2^ = 86%
Coptidis + conventional vs. conventional (n = 3)	−1.44 [−2.53, −0.35]	*p* < 0.00001, I^2^ = 93%
Total	−0.92 [−1.18, −0.66]	*p* < 0.00001, I^2^ = 83%

### Appendix B.5. Homeostatic Model Assessment of Insulin Resistance

Six trials evaluated the adjunctive use of Coptidis-containing formulations in combination with antidiabetic medications compared with antidiabetic medications alone. Three trials compared Coptidis-containing formulations plus GLP-1 RAs with GLP-1 RAs alone. Two trials assessed Coptidis-containing formulations combined with metformin versus metformin monotherapy, while a single trial compared Coptidis-containing formulations plus conventional treatment with conventional treatment alone. The corresponding results are summarized in Table A8 and Figure A5.

**Table A8:** Meta-analysis summary of Homeostatic model assessment of insulin resistance.

	Std. Mean Difference	Heterogeneity
Coptidis + antidiabetic vs. antidiabetic (n = 6)	−0.95 [−1.24, −0.66]	*p* = 0.03, I^2^ = 59%
Coptidis + GLP-1 RA vs. GLP-1 RA (n = 3)	−0.93 [−1.31, −0.56]	*p* = 0.04, I^2^ = 68%
Coptidis vs. metformin (n = 2)	−0.58 [−0.95, −0.22]	*p* = 0.84, I^2^ = 0%
Coptidis + conventional vs. conventional (n = 1)	−0.85 [−1.26, −0.44]	Not applicable
Total	−0.88 [−1.05, −0.70]	*p* = 0.03, I^2^ = 49%

### Appendix B.6. Total Cholesterol

Six trials evaluated the adjunctive use of Coptidis-containing formulations in combination with antidiabetic medications compared with antidiabetic medications alone. Two trials assessed Coptidis-containing formulations combined with metformin versus metformin monotherapy, while three trials compared Coptidis-containing formulations plus conventional treatment with conventional treatment alone. The corresponding results are summarized in Table A9 and Figure A6.

**Table A9:** Meta-analysis summary of total cholesterol.

	Std. Mean Difference	Heterogeneity
Coptidis + antidiabetic vs. antidiabetic (n = 6)	−1.09 [−1.48, −0.71]	*p* = 0.002, I^2^ = 74%
Coptidis vs. metformin (n = 2)	0.05 [−0.31, 0.41]	*p* = 0.64, I^2^ = 0%
Coptidis + conventional vs. conventional (n = 3)	−0.53 [−1.04, −0.01]	*p* = 0.02, I^2^ = 73%
Total	−0.73 [−1.09, −0.37]	*p* < 0.00001, I^2^ = 84%

### Appendix B.7. Triglycerides

Six trials evaluated the adjunctive use of Coptidis-containing formulations in combination with antidiabetic medications compared with antidiabetic medications alone. Two trials assessed Coptidis-containing formulations combined with metformin versus metformin monotherapy, while three trials compared Coptidis-containing formulations plus conventional treatment with conventional treatment alone. The corresponding results are summarized in Table A10 and Figure A7.

**Table A10:** Meta-analysis summary of triglycerides.

	Std. Mean Difference	Heterogeneity
Coptidis + antidiabetic vs. antidiabetic (n = 6)	−1.21 [−1.91, −0.50]	*p* < 0.00001, I^2^ = 92%
Coptidis vs. metformin (n = 2)	−0.84 [−1.22, −0.47]	*p* = 0.55, I^2^ = 0%
Coptidis + conventional vs. conventional (n = 3)	−0.71 [−1.42, 0.00]	*p* = 0.0001, I^2^ = 85%
Total	−1.00 [−1.42, −0.58]	*p* < 0.00001, I^2^ = 88%

### Appendix B.8. High-Density Lipoprotein Cholesterol

Four trials evaluated the adjunctive use of Coptidis-containing formulations in combination with antidiabetic medications compared with antidiabetic medications alone. Two trials assessed Coptidis-containing formulations combined with metformin versus metformin monotherapy, while three trials compared Coptidis-containing formulations plus conventional treatment with conventional treatment alone. The corresponding results are summarized in Table A11 and Figure A8.

**Table A11:** Meta-analysis summary of high-density lipoprotein cholesterol.

	Std. Mean Difference	Heterogeneity
Coptidis + antidiabetic vs. antidiabetic (n = 4)	0.85 [0.59, 1.10]	*p* = 0.29, I^2^ = 20%
Coptidis vs. metformin (n = 2)	0.89 [0.16, 1.62]	*p* = 0.05, I^2^ = 73%
Coptidis + conventional vs. conventional (n = 3)	1.07 [0.48, 1.65]	*p* = 0.01, I^2^ = 77%
Total	0.93 [0.69, 1.17]	*p* = 0.02, I^2^ = 56%
